# Optimisation and Validation of an Induced Membrane Technique Model to Assess Bone Regeneration in Rats

**DOI:** 10.1155/term/7357277

**Published:** 2025-04-21

**Authors:** Renaud Siboni, Johan Sergheraert, Lea Thoraval, Christine Guillaume, Sophie C. Gangloff, Xavier Ohl, Julien Braux, Frédéric Velard

**Affiliations:** ^1^Biomaterial and Inflammation in Bone Site Laboratory (EA 4691 Bios), University of Reims Champagne Ardenne, 51 Rue Cognacq Jay, Reims 51100, France; ^2^Department of Orthopaedic Surgery, Reims University Hospital, Hôpital Maison Blanche, 45 Rue Cognacq-Jay, Reims 51092, France; ^3^UFR Médecine, University of Reims Champagne Ardenne, 51 Rue Cognacq Jay, Reims 51100, France; ^4^Department of Dental Surgery, Reims University Hospital, Hôpital Maison Blanche, 45 Rue Cognacq-Jay, Reims 51092, France; ^5^UFR Pharmacy, University of Reims Champagne Ardenne, 51 Rue Cognacq Jay, Reims 51100, France

## Abstract

**Background:** The induced membrane (IM) preclinical models have been described in small animals, but few studies have looked at bone regeneration achievement. The optimisation and validation of such a preclinical model, considering the results obtained after the use of biomaterials as a substitute for bone grafting, could lead to simplifying the surgical procedure and enhance the clinical results.

**Methods:** An in vivo model of the IM technique was developed on the femur of Lewis rats after a 4-mm critical bone defect stabilised with an osteosynthesis plate. A first optimisation phase was performed by evaluating different osteotomy methods and two different osteosynthesis plate sizes. The efficiency of the model was evaluated by the failure rate obtained 6 weeks after the first operative time. Thereafter, bone regeneration was evaluated histologically and radiologically at 24 weeks to confirm the critical nature of the bone defect (negative control), the effectiveness of the IM with a syngeneic bone graft (positive control) and the possibility of using a biomaterial (GlassBone Noraker) in this model.

**Results:** Sixty-three rats were included and underwent the first surgical step. Nineteen rats subsequently underwent the second surgical step. The results obtained led to select piezotomy as the best osteotomy technique and 1-mm-thick plates with 2.0-mm-diameter screws as osteosynthesis material. Twenty-four weeks after the second surgical step, solely the group with both surgical steps and a syngeneic bone graft showed complete ossification of the bone defect. In contrast, the group without a graft did not present a suitable ossification, which confirms the critical nature of the defect. IM produced an incomplete bone regeneration using GlassBone alone.

**Conclusions:** A piezotome osteotomy with an osteosynthesis plate of sufficient stiffness is required for this two-stage bone regeneration model in rats. The 4-mm bone defect is critical for this model and suitable for biomaterial evaluation.

## 1. Introduction

A critical bone defect has been clinically defined as being 2 to 2.5 times the bone diameter in length [1] and not allowing spontaneous bone healing [2]. Such defects may arise from infection, tumours or trauma [3], causing significant suffering to the patient, with a socio-economic burden of 3 billion US dollars each year and affecting 2 million people worldwide [4]. In humans, when long bone defects do not exceed 4–5 cm, an autologous bone graft is often sufficient to achieve bone healing [5, 6]. For larger defects, a simple bone graft seems not to suffice due to the excessive resorption of the graft [7]. In such cases, techniques such as osteogenic distraction (Ilizarov technique [8]), vascularised graft [9] or the induced membrane (IM) technique [10] are needed [11].

The IM technique is the most recently developed. It was first described by Alain-Charles Masquelet in 1986, and its clinical value was first described in 2000 and 2003 [12]. It is a two-step procedure: In the first step, the bone is stabilised using external or internal fixation, followed by the placement of a polymethyl methacrylate (PMMA) spacer in a freshly generated bone defect. After soft tissue closure, a 4- to 6-week period allows the foreign body reaction to produce a membrane enveloping the cement spacer. In the second stage, new surgical access is made to preserve this membrane; the PMMA is removed, and the bone defect is filled with an autologous bone graft, with the IM preserved and closed around it [10, 13]. Several clinical studies have evaluated its efficacy [12], highlighting that more than two surgical steps may be required to obtain suitable results, as well as the need to use a high amount of autologous bone graft, which the patient often does not have enough of [11]. The use of biomaterials that improve bone regeneration could simplify this procedure.

Basic scientific studies have been conducted to understand this procedure [12]. The presence of a cement spacer results in a foreign-body inflammatory reaction leading to the creation of an organised pseudosynovial membrane, which is rich in inductive molecules [14]. The cells retrieved from the IM express markers of bone marrow-derived mesenchymal cells have higher levels of growth factors than periosteum cells. Preclinical models have been described in goat [12], sheep [1, 15], rabbit [14] and rat [16–27], but few studies have evaluated bone regeneration after these two surgical steps, especially in small animals [16]. To assess the efficacy of biomaterials or bone tissue engineering techniques, critical femoral defect models are often considered the gold standard. However, such interventions are time-consuming and expensive and present high technical barriers [28].

Since they have bone mechanics similar to those of humans [29, 30], the rat is an animal model often used for bone regeneration research. The Lewis strain of rats allows for long-term CT radiological follow-up due to its limited growth. The inbreeding of this strain enables the use of a syngeneic graft. To refine the Lewis rat model of the IM procedure, we propose to (1) perform the best osteotomy combined with plate osteosynthesis; (2) assess bone consolidation in critical femoral bone defects; and (3) evaluate a biomaterial's capacity for bone consolidation in this model.

## 2. Materials and Methods

### 2.1. Animals

We made an effort to uphold the replacement, refinement and reduction of animals and their suffering. Milestones for stopping or continuing experimental conditions were determined so as not to systematically engage animals in a procedure that would not be operable. Concrete endpoints were defined to ensure animal welfare. After approval by the animal experimentation ethics committee (APAFIS N° 2017031403204417), we used male rats (LEWIS—Janvier Laboratories), 12 weeks of age, allowing us to work on mature long bones of a size that would facilitate surgical approaches and placement of orthopaedic material, following the model already validated by Gouron et al. [17]. The animals were kept alone in plastic cages in an accredited animal facility under a controlled environment (temperature 21°C (± 1°C), humidity 55% ( ± 10%), ventilation air volume renewed 20 times per hour, alternating 12-h light/dark cycles). The rats had free access to water and a standard laboratory rodent diet. Clinical, radiological and histological evaluation was blinded.

### 2.2. Surgical Technique (See Figure S1 in the Supporting Information)

The surgical procedure was performed after 10 days of acclimation. The anaesthesia protocol was performed by gas induction within an induction cage (isoflurane 5%—Zoetis). The animals were maintained under anaesthesia by mask insufflation of 2.5% isoflurane with 1.8 L/min oxygen. Intraoperative analgesia was through the subcutaneous injection of meloxicam (Metacam—Boehringer—0.2 mg per kg of body weight) coupled with intraoperative antibiotics and anti-inflammatory prophylaxis (Cortexiline—Merial—corresponding to 11.4 mg of benzylpenicillin, 12 mg of neomycin and 0.4 mg of methylprednisolone per kg body weight).

#### 2.2.1. First Stage of Surgery

After povidone–iodine decontamination, a skin incision was made on the outer surface of the right thigh, followed by dissection of the vastus lateralis and biceps femoris muscles down to the femoral shaft. A four-hole titanium osteosynthesis plate (MEDARTIS) was placed and maintained by four bicortical screws after predrilling the bone with a motor (30,000 rpm) using a suitable drill bit (1.0/1.6 mm). A 4-mm bone defect was required to induce a critical defect [31]. The bone defect was left empty (Empty condition) or filled with syngeneic bone material (Syngeneic condition) or a cement spacer (PMMA + gentamicin) (PMMA condition) made with a cylindrical preform.

The muscle and skin were sutured with an absorbable suture (Vicryl Rapide 4.0). An in vivo radiological examination with an X-ray microscanner (Skyscan 1076, Bruker) was performed at 14 days and 6 weeks to detect loosening of the osteosynthesis. Animals with primary fixation failures were euthanised.

#### 2.2.2. Second Operative Step

It is performed only for two-staged conditions. After povidone–iodine decontamination, the initial incision was repeated. The generated IM was opened, the PMMA spacer was extracted with gouge forceps, and a graft consisting of previously ground syngeneic femoral bone (Ultra-Turrax, IKA) was placed or the space was filled with bioactive glass (GlassBone—Noraker) or left free. An absorbable running suture (Vicryl Rapide 4.0) of the IM was applied around the graft. Sutures of the muscular and cutaneous planes were then made with absorbable thread (Vicryl Rapide 4.0). Full weight-bearing was achieved for all the rats.

After each operation, twice-daily clinical and daily weight monitoring was performed for the first 72 h with analgesic treatment for the first 6 days (0.2 mg of metacam per kg of body weight once a day subcutaneously). Thereafter, clinical and weight monitoring was performed weekly throughout the study. Humane endpoints were defined beyond which the experimentation had to be stopped. This score was calculated and adapted based on the Morton and Griffiths scale score (M & G) [32] (see Figure S2 in the Supporting Information). A score ≥ 9/15 or a displacement of the osteosynthesis material between two CT radiological controls was defined as an endpoint.

**Figure 1:**
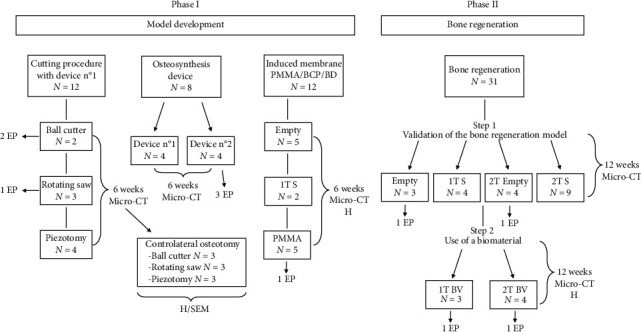
Flowchart of the study. 1T: one-step surgery; 2T: two-step surgery; BCP: best cutting procedure; BD: best device; BV: bioactive glass; EP: number of rats reaching the endpoint; H: histology; micro-CT: microcomputed tomography; PMMA: polymethyl methacrylate; S: syngeneic graft; SEM: scanning electron microscopy.

To minimise the number of animals used as much as possible, a ‘go/no-go' type of experimentation was performed to choose the most suitable osteotomy mode, use the appropriate osteosynthesis material and confirm the induction of an IM (see Figure 1).

### 2.3. Improvement of the Osteotomy Procedure

Three osteotomy modalities were evaluated under abundant irrigation: a tungsten carbide ball bur (08—Komet) mounted on a dental surgical counter angle (Implant Medplus—W&H), a diamond saw disc for intraoral bone harvesting (Frios MicroSaw-Dentsply) and a piezotome (Piezotome solo + BS1-V insert—Acteon). Four rats were randomly assigned to three groups according to the osteotomy type. In each group, the lesion was stabilised with an osteosynthesis plate (MEDARTIS thickness 0.6 mm, screw diameter 1.5 mm, length 4 mm). A 4-mm defect was created in each group. Six weeks later, the rats were euthanised to harvest the operated femur for CT analysis of the integrity of the osteosynthesis plates. The opposite femur was also harvested to obtain one of the 3 osteotomy sections for histological studies by optical microscopy and SEM.

### 2.4. Evaluation of the Osteosynthesis Material

This experiment was conducted on two groups of four rats to evaluate two different sizes of four-hole titanium plates with four screws (MEDARTIS) and a bone defect of 4 mm made by the previously validated osteotomy technique. The first group had a 0.6-mm-thick plate with 1.5-mm-diameter, 5-mm-long screws; the second group had a 1.0-mm-thick plate with 2.0-mm-diameter, 6-mm-long screws. An in vivo CT scan was performed 14 days postoperatively to look for early loosening of the material, as well as at the terminal time point at six weeks.

### 2.5. Evaluation of the IM

This last protocol was performed by choosing the most relevant techniques based on the above experiments. To evaluate the generation of the IM, three groups were created. The first group (PMMA, *n* = 5) had a cement spacer. A negative control group with a bone defect had no spacer (Empty, *n* = 5). A second group had a syngeneic graft placed into the bone defect (1T-S, *n* = 2). A histological evaluation of the IM was performed at 6 weeks, combined with a CT scan for all three groups after the rats were sacrificed.

### 2.6. Final Protocol

#### 2.6.1. Assessment of Bone Regeneration

Four groups were formed: The Empty control group (*n* = 4) to assess the critical nature of the defect when left free, the 1T-S group to evaluate bone consolidation when there was a syngeneic graft without the IM technique (*n* = 4), the 2T Empty control group to evaluate the critical nature of the defect when left free with the IM technique (*n* = 5) and the 2T-S group to evaluate bone consolidation with a syngeneic graft and the IM technique (*n* = 9). The Empty and 1T-S groups underwent only one surgical step, and the other two groups underwent two surgical steps spaced 6 weeks apart. A CT radiological evaluation up to 24 weeks after the last step was performed in all 4 groups, along with a qualitative histological analysis at 24 weeks.

#### 2.6.2. Evaluation of the Biomaterial

Bone regeneration was evaluated in this IM model with the use of a biomaterial (GlassBone—Noraker), either alone in the bone defect (1T-BV group *n* = 4) or in combination with the IM during the second stage (2T-BV group, *n* = 5). Bone consolidation was assessed for up to 24 weeks on CT images after the second stage, along with a qualitative histological analysis at 24 weeks.

#### 2.6.3. Evaluation Methods—SEM

Samples dedicated for osteotomy analysis were fixed in 2.5% glutaraldehyde (Sigma-Aldrich) (in 1X PBS, Life Technologies) for 1 h at room temperature. They were rinsed with distilled water twice for 10 min. The samples were dehydrated by successive passages of 10 min in alcoholic solutions of increasing concentrations of 50°, 70° and 90° and finally with two baths in absolute ethanol (Charbonneaux Brabant). Decuturization was carried out thereafter through a five-minute bath of an equivalent volume of absolute ethanol and hexamethyldisilazane (Sigma Aldrich) at room temperature and then by another bath of pure hexamethyldisilazane, always under a fume hood and at room temperature, until complete evaporation. The metallization was achieved with palladium gold in a cathodic evaporator (JEOL ION SPUTTER JFC-1100) at 8 mA and 1200 V. The prepared samples were analysed in a JEOL 5400 LV SEM.

#### 2.6.4. Evaluation Methods—Histology

For bone tissue analysis, the specimens were dehydrated in a graded series of alcohol and embedded in spurs. Slices in the longitudinal direction of the implant were cut with a laser microtome (TissueSurgeon, LLS ROWIAK GmbH, Hannover, Germany) and stained with Masson's trichrome. The slice thickness was 10 μm. Scanning was performed using an Olympus VS120 imager, and detailed photographs were acquired using an Axiovert 200M digital microscope (Zeiss) at × 20 (AxioVision) magnification. Samples were evaluated qualitatively in terms of structure, the reaction of surrounding tissue and bone formation.

For in situ IM analysis, samples were fixed with 10% formalin (VWR) and then decalcified through baths in ethylene diamine tetra acetic acid (EDTA 0.5 M, Sigma-Aldrich in 1 × PBS) at room temperature and under agitation for 21 days with changes in the decalcification solution two times per week. Alcoholic dehydration via two 20 min baths in 95° alcohol and three 20 min baths in 100° alcohol was then performed. Two 20-min toluene (VWR) baths were then done, followed by three one-hour paraffin baths at 56°C. Inclusion was performed by dipping the samples into paraffin heated to 56°C. Five-micron-thick sections were made with a microtome (MICROM HM 355S) and then spread onto a slide. Masson's trichrome staining was performed in successive baths: three toluene baths for 5 min, a 100° alcohol bath for 5 min, two 95° alcohol baths for 3 min, a distilled water bath for 5 min, a hemalun bath (Prolabo) for 8 min, a running water bath for 5 min, a 3-min fuchsin bath (Merck), a 3-min distilled water bath, a 5-min phosphomolybdic acid bath, a 5-min aniline blue bath (Merck), a 5-min acetic water bath, two 100° alcohol baths, and then two toluene baths. The prepared samples were observed with a Zeiss Axiovert 200M optical microscope under the × 20 objective (AxioVison software).

#### 2.6.5. Evaluation Methods—X-ray Microtomography

Tomographic evaluations were performed on a Skyscan 1076 X-ray microscanner (Bruker) under an accelerating voltage of 100 kV and a tube current of 0.100 mA. A 0.025-mm titanium filter was used. The voxel sizes were 35.8 × 35.8 × 35.8 μm (*x*, *y*, *z*) for longitudinal evaluation and 17.9 × 17.9 × 17.9 μm (*x*, *y*, *z*) for terminal evaluation at 24 weeks. The longitudinal evaluation was focused on the bone defect to avoid artefacts related to a cutting defect or bone consolidation along the plate. The final evaluation focused on the space between the two central screws of the osteosynthesis assembly. The 3D images were reconstructed using NRecon GPU software (Bruker) and reoriented using Dataviewer software. After 3D reconstruction and reorientation, regions of interest were defined using the mean value of the bone densities outside the treated region, collected in each scan, after manual segmentation. Two types of regions of interest were created: a parallelepiped measuring 4.5 mm × 4.5 mm × 2 mm, manually centred into the defect to assess the volume of bone regenerated in the centre of the defect, and a wider region, measuring 4.5 mm × 4.5 mm × 8 mm, manually centred into the defect to assess the volume of bone regenerated in the whole defect. Osteosynthesis systems were segmented and subtracted before analysing bone volumes. At this time, the bone volume of the contralateral (CL) healthy femur of each rat was measured for comparison with the operated area. Bone volumes were calculated using Fiji software [33].

### 2.7. Statistical Analysis

Statistical analysis was performed with StatXact7 software (Cytel Studio). Quantitative variables are visualised as whisker box plots presenting the median and the interquartile and interdecile spaces of the data series. The nonparametric Wilcoxon–Mann–Whitney exact test was used to compare the quantitative variables, with a significance threshold of *p* < 0.05. We used nonparametric statistics because could not assess for a normal distribution of the assessed variables.

## 3. Results and Discussion

Eleven rats reached the endpoint requiring removal from the experiment. The 1 TS group from the IM evaluation was reduced from five to two rats after initial evaluation of the IM at the end of the first experimental run. The results for the first two rats in this group showed no membrane in contact with the syngeneic graft at 6 weeks (Figure 1).

Sixty-three rats were ultimately included and underwent the first surgery. Nineteen rats underwent the second surgical step at 6 weeks. Clinical follow-up was conducted throughout the various procedures for each rat (see Figures S3–S6 in the Supporting Information).

### 3.1. Is the use of Piezotome Osteotomy Combined With Plate Osteosynthesis Suitable?

#### 3.1.1. Osteotomy Procedure

**Figure 2:**
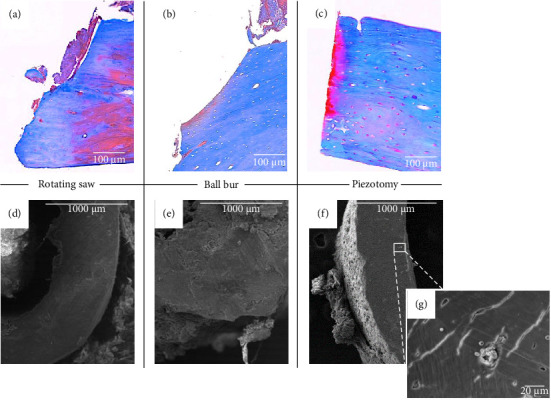
Images from the upper row (a–c) show the histological assessment of the cut bone extremity (Masson's trichrome staining, Zeiss Axiovert 200M, magnification × 20, scale bar = 100 μm). Images from the lower row (d–g) show scanning electron microscopy (JEOL 5400 LV) (15 kV, magnification × 50 (a–f), scale bar = 1000 μm; magnification × 1000 (g), scale bar = 20 μm). Burned appearance of bone cuts in conditions a, b, d and e. Permeable appearance of bone cuts with the piezotome (c and f). At higher magnification, the presence of a permeable Haversian canal with red blood cells inside (g).

Altogether, 12 rats were operated on and followed up for 6 weeks: four with piezotome osteotomy, four with a ball bur and four with a rotating saw. Three rats were euthanised during the surgical procedure (two from the ball cutter condition and one from the rotating saw condition) because of an intraoperative fracture during the fixation of one of the screws. At the 6-week CT radiological follow-up, all the rats showed displacement of the material (see Figure S7 in the Supporting Information). Piezotomy allowed the most precise osteotomy, without trauma to the soft tissues or the plate. On histological analysis, the bone cuts were irregular when we used a saw and bur, with a burnt appearance of the bone edges. With piezotomy, the sections were cleaned with a permeable aspect of the bone edges. These judgements were supported by electron microscopy analysis, which revealed a permeable appearance of the Haversian channels with piezotomy and closure for the other two cutting techniques (see Figure 2).

#### 3.1.2. Osteosynthesis Material

Eleven rats underwent piezotomy osteotomy. Three rats had an intraoperative fracture when the osteotomy was performed, and these rats were euthanised preoperatively. Material number two (1.0-mm-thick plate with 2.0-mm-diameter, 6-mm-long screws) was the only construct to remain in place during follow-up. All rats with thinner hardware (0.6-mm-thick plate with 1.5-mm-diameter, 5-mm-long screws) had hardware displacement at 6 weeks (see Figure S8 in the Supporting Information).

Therefore, the intraoperative failure rate was 100% for the thin material and 18.75% for the thicker material.

### 3.2. Is it Possible to Assess Bone Consolidation Through the IM Technique in This Critical Model of Bone Defects in the Femurs of the Lewis Rat?

#### 3.2.1. IM

**Figure 3:**
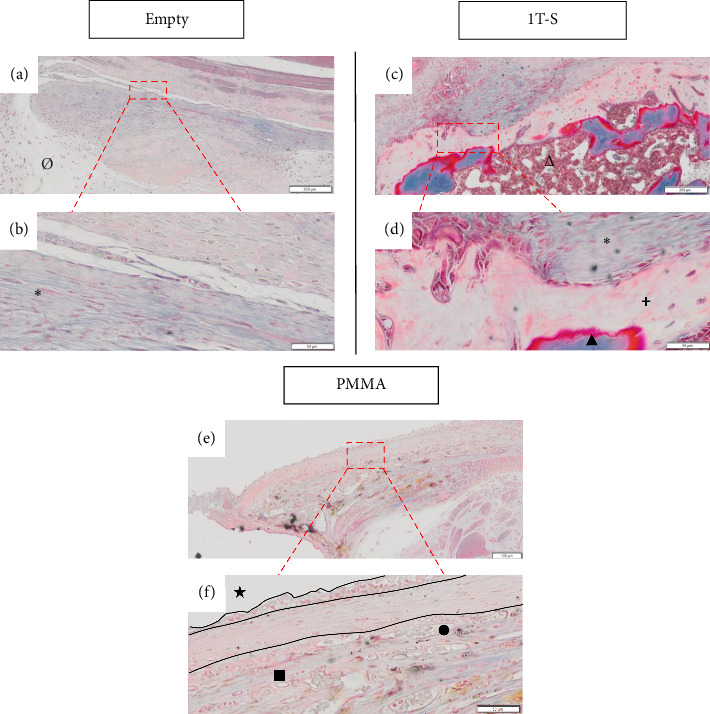
IM formation was assessed qualitatively on histological sections stained with Masson's trichrome ((a, c and e): Objectif × 10, scale bar = 100 μm (b, d and f) Objective × 40, scale bar = 50 μm). Bone defect (∅), syngeneic graft (∆), PMMA (★), muscle (∎), vessels (●), bone (▲), fibrous tissue (^∗^) and soft callus (✚) were detected. 1T: one-step surgery; PMMA: polymethyl methacrylate; S: syngeneic graft.

Twelve rats underwent a single surgical step: five rats in the PMMA group, five rats in the Empty group, and two rats in the 1T-S group. One rat in the PMMA group was euthanised due to displacement of the material during longitudinal CT radiological control. On macroscopic histological analysis, only the PMMA group had a foreign body reaction, forming an IM organised in three layers: an inner layer in contact with the PMMA with a high cell density, an intermediate layer with a lower cell density and a peripheral layer in contact with the muscle with the presence of vessels. In the Empty and 1T-S groups, disorganised fibrous tissue was found (see Figure 3).

#### 3.2.2. Bone Healing—Step 1

**Figure 4:**
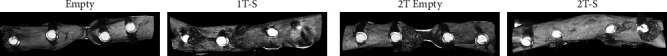
3D reconstructions (a and b) of X-ray microtomography images of rat femurs operated on and filled, or not, with syngeneic material (Skyscan 1076 Brucker—Voxel 17.9 μm × 17.9 μm × 17.9 μm). (a) Empty and syngeneic grafts with only the first stage of the procedure. Persistent bone nonunion at 12 weeks. (b) Empty and syngeneic grafts with the two stages of the procedure. Persistent bone nonunion with the Empty condition and bone healing with the syngeneic graft condition. 1T: one-step surgery; 2T: two-step surgery; S: syngeneic graft.

Twenty-two rats underwent surgery. Eight rats underwent only the 1st surgical step: four rats from the Empty group and four rats from the 1T-S group. Fourteen rats underwent both surgical steps: five in the 2T-E group and nine in the 2T-S group. Two rats were euthanised due to displacement of the osteosynthesis material after the second surgical step: one rat in the Empty group and one rat in the 2TE group. At the final CT radiological check-up, only the group with the IM (2T-S) had achieved consolidation: seven consolidated rats and one nonconsolidated rat with hardware failure. In the other three groups, consolidation was not observed at the last follow-up. However, bone regeneration was higher in the 1T-S group, with the persistence of bone nonunion (see Figure 4).

### 3.3. Is it Possible to Assess a Biomaterial's Capacity for Bone Consolidation in this Model?—STEP 2

**Figure 5:**
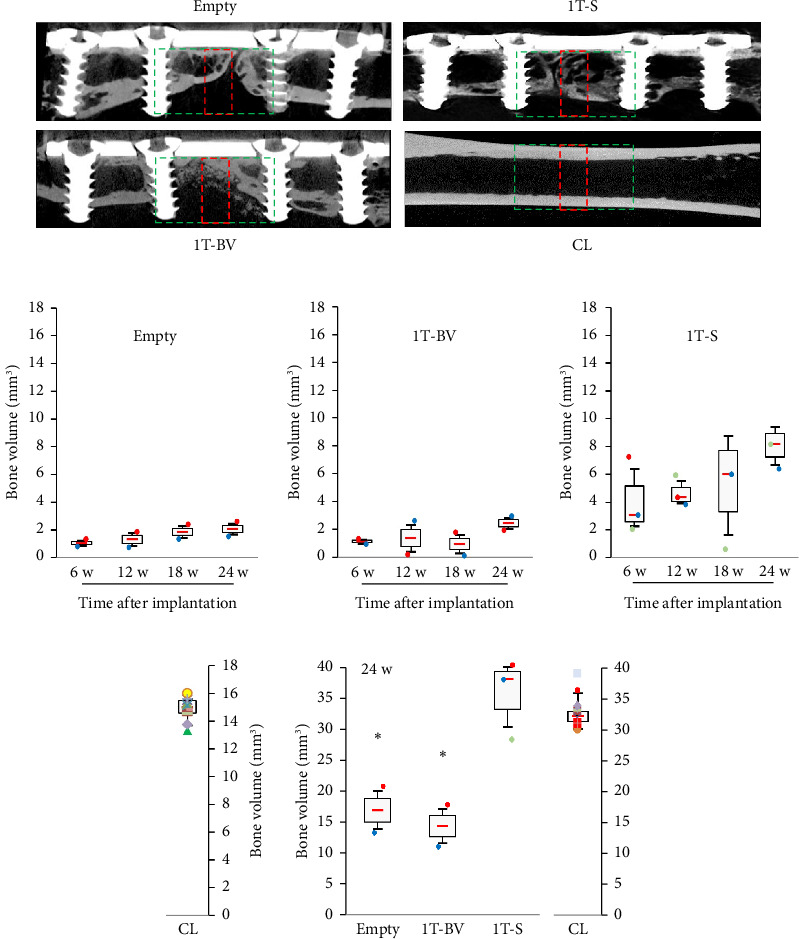
Medial section of 3D reconstructions of X-ray microtomography images of rat femurs operated and filled, or not, with the material of interest with only the first surgical step. Skyscan 1076 Brucker. (a) The red rectangle illustrates the results of (b–e) the quantification (mm^3^) of the amount of mineral in the region of interest (Voxel 35.8 × 35.8 × 35.8 μm) at 6, 12, 18 and 24 weeks in the (b) Empty, (c) 1T-BV (Glassbone with a single surgical step), (d) 1T-S (syngeneic bone with a single surgical step) and (e) CL (contralateral femur). (f) Quantification (mm^3^) of the amount of mineral in the green region of interest (Voxel 17.9 × 17.9 × 17.9 μm) at 24 weeks, for all conditions and contralateral femur, ^∗^*p* < 0.05 vs. contralateral. Red bar represents the median, and each mark indicates a rat.

Nine rats underwent surgery. Four rats were included in the 1T-BV group, and five in the 2T-BV group. One rat in the 1T-BV group was euthanised due to material failure during follow-up, and one rat in the 2T-BV group died during anaesthesia at the CT radiological check-up after the second surgical step. At CT radiological follow-up, consolidation was not achieved in either group, with resorption of the biomaterial. At 24 weeks in the one-step surgery group, the amount of bone in the bone defect was greatest in the 1T-S condition, followed by the CL, 1T-BV and Empty conditions, with mean values equal to 35.6 mm^3^ [28.4–40.4], 32.6 mm^3^ [29.4–38.3], 17.1 mm^3^ [13.2–20.7] and 14.4 mm^3^ [11.0–17.8], respectively (see Figure 5(f)). No significant difference was observed between the 1T-S and 1T-BV conditions. No significant difference was observed in the Empty condition compared to the 1T-S and 1T-BV conditions. Compared to the CL, the Empty (*p*=0.032) and 1T-BV conditions were significantly different (*p*=0.032), but not the 1T-S condition.

**Figure 6:**
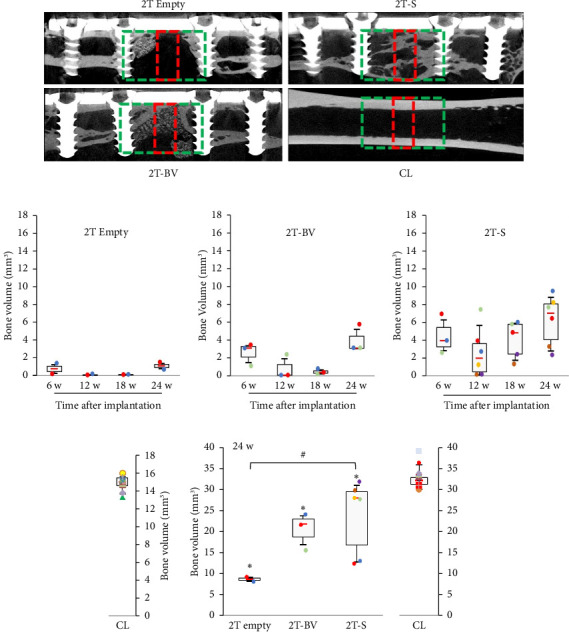
Medial section of 3D reconstructions of X-ray microtomography images of rat femurs operated and filled with PMMA for all conditions at the first surgical time and then reoperated to remove the PMMA. The defect was refilled, or not, with the material of interest at 24 weeks. Skyscan 1076 Brucker. (a) The red rectangle illustrates (b–e) quantification (mm^3^) of the amount of mineral in the region of interest (Voxel 35.8 × 35.8 × 35.8 μm) at 6, 12, 18 and 24 weeks for the following conditions: (b) 2T Empty (with the two surgical steps), (c) 2T-BV (GlassBone with the two surgical steps) and (d) 2T-S (syngeneic bone with the two surgical steps), (e) CL (contralateral femur). (f) Quantification (mm^3^) of the amount of mineral in the green region of interest (Voxel 17.9 × 17.9 × 17.9 μm) at 24 weeks, for all conditions, ^∗^*p* < 0.05 vs. contralateral femur, ^#^*p* < 0.05 vs. 2T Empty. Red bar represents the median, and each mark indicates a rat.

**Figure 7:**
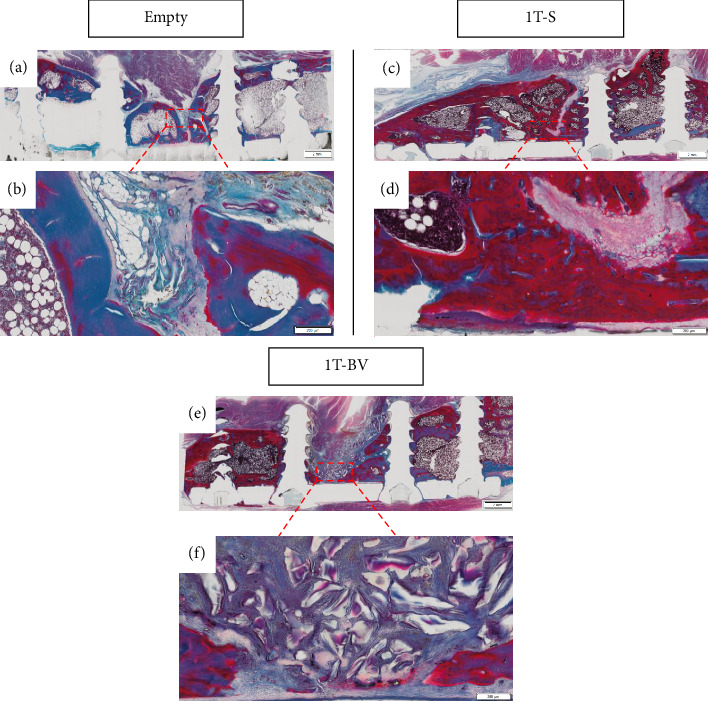
Bone formation was assessed qualitatively on histological sections stained with Masson's trichrome using optical microscopy (Olympus VS120 imager; (a, c and e) Objective × 2, scale bar = 2 mm (b, d and f) Objective × 20, scale bar = 200 μm). In all conditions, there is no bone consolidation. In the 1T-BV (Glassbone with a single surgical stage) condition, areas of ossification are observed in contact with the bioactive glass, with the bone defect being invaded by scar tissue. 1T: one-step surgery; BV: bioactive glass; S: syngeneic graft.

**Figure 8:**
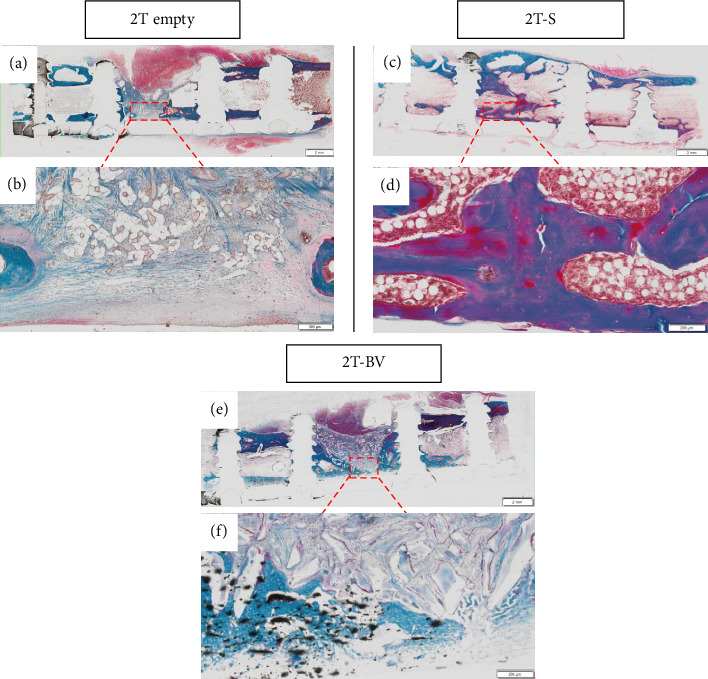
Bone formation was assessed qualitatively on histological sections stained with Masson's trichrome via optical microscopy ((a, c and e) Objective × 2, scale bar = 2 mm (b, d and f) Objective × 20, scale bar = 200 μm). Only the 2T-S condition achieves complete consolidation of the bone defect. The 2T Empty (with the surgical stages) condition remains empty, with the presence of fibrous tissue and lipid inclusions. The 2T-BV (Glassbone with the two surgical stages) condition shows the persistence of the biomaterial with ossifications observed adjacent to it. This condition appears to have less scar tissue inclusion and better preservation of the biomaterial, potentially explained by the presence of the membrane. 2T: two-step surgery; BV: bioactive glass; S: syngeneic graft.

For two-step surgeries, the greatest amount of bone in the area of interest was found in the CL, followed by the 2T-S, 2T-BV and 2T Empty conditions, with mean values equal to 32.6 mm^3^ [29.4–38.3], 24.0 mm^3^ [12.5–32.0], 21.0 mm^3^ [15.7–24.3] and 8.8 mm^3^ [8.2–9.3], respectively (see Figure 6(f)). This difference was not significant between the 2T-S and 2T-BV conditions. Compared to the 2T Empty condition, the difference was significant in the 2T-S condition (*p*=0.046) but not significant in the 2T-BV condition. Compared with the CL condition, the 2T-S (*p*=0.007), 2T-BV (*p*=0.011) and 2T Empty conditions were significantly different (*p*=0.032) (see Figures 5(a), 5(b), 5(c), 5(d), 5(e), 6(a), 6(b), 6(c), 6(d), 6(e)). Consolidation was complete in the 2T-S group and absent in all other conditions. Biomaterial resorption appeared to be greater in the 1T-BV condition than the 2T-BV condition (see Figures 5, 6, 7, 8). On histological evaluation, this biomaterial resorption as well as lipid inclusions were more marked in the 1T-BV group (see Figure 7) than in the 2T-BV condition (see Figure 8). Areas of ossification were present when in contact with the biomaterial in both groups.

The bone regeneration ability of a biomaterial in a preclinical critical bone defect model in rats can be evaluated with the IM technique. The major results of this study were as follows: (1) a 1.0-mm-thick plate and screw 2.0 mm in diameter and 6 mm in length must be used; (2) a 4-mm bone defect in the femur was enough to be critical; and (3) piezotome osteotomy was the most suitable intervention for this model.

This study was limited by the small number of subjects. This was related to the design of our study, which was established to develop a preclinical animal model associated with the constraint of replacement, refinement and reduction of animals due to ethical concerns. As it is a preclinical model, this regeneration model is not directly applicable to humans, but it can provide indispensable results in the evaluation of new biomaterials for bone regeneration.

Several animal models have been described and used to characterise the IM technique [15, 17]. The use of large animal models allows the mechanical constraints of loading to be taken into account, and the dimensions are convenient for evaluating implants or bone substitutes [15, 34]. The disadvantages of these models are their high cost, low availability and ethical concerns [29]. A rat femur model is more valuable than a large animal model; in fact, the use of a rat femur model is reproducible, standardised, fast and less expensive [29]. Most studies [17–21, 23] have used this model to characterise the IM, but few have evaluated bone regeneration [22, 24–26]. Only the studies by Nau et al. [26] and Bosemark et al. [24] investigated a biomaterial in their models. The bone defects used in these studies were 6 and 10 mm long, respectively, and the animals were Sprague–Dawley rats. In our model, a defect of 4 mm was enough to induce a critical defect in a Lewis rat model [31]. Our choice was for the inbred Lewis strain, allowing us to perform a syngeneic transplant. Moreover, this strain has a lower growth rate than others and is suitable for the scanner we used for in vivo monitoring.

The type of osteotomy used preferentially in the rat models described was the reciprocating saw [20, 22, 23, 25] or Gigli [21, 24, 26]. In this study, the osteotomy of choice was piezotomy. Too many subjects were lost with the diamond bur and the saw, despite good precision, and the risk of damage to the osteosynthesis material and the peripheral soft tissues was high. Piezotomy enables a precise procedure due to the control of haemostasis by cavitation [35] and the absence of damage to the surrounding tissues of the bone by cutting only the mineralised substances [36]. Moreover, histological results showed a defect suitable for bone regeneration with increased lamellar separation of the bone, accentuating the communication with the Haversian canals, as found in the study by Horton et al. [37].

The osteosynthesis device must be rigid enough [38, 39] to allow immediate support of the bone defect. Several methods of support have been described, such as the use of an external fixator, an intramedullary nail and an osteosynthesis plate. The external fixator is easy to use but has a high risk of infection at the level of the pins and should preferably be used in studies that only characterise the IM [29]. The intramedullary nail is also easy to use, but at risk of fracture during placement, it requires heavier rats and is the most expensive of the three methods [18, 19, 29]. The osteosynthesis plate allows optimal displacement reduction, has little influence on bone defects and provides ample space for the use of a biomaterial. This method is less expensive than the one with the intramedullary nail but requires additional surgical skills for its use [29]. For these reasons, and because of the lesser space required by the external fixator, we chose a nonlocking osteosynthesis plate, which is used in current orthopaedic surgery practice. It must be fairly stiff for this model to be viable. For rats weighing 350 g–400 g, a 1-mm-thick plate with four screws on either side of the defect with a diameter of at least 1.1 mm is necessary, even for defects up to 10 mm [20–23, 26, 27]. To reduce artefacts from osteosynthesis material in radiological analysis, radiolucent materials can be used [25]. However, we did not select this type of material due to its limited use in clinical practice.

At the last follow-up, the rats in the 2T group had consolidated their bone defects. Bone volume seemed to be greater in the 1T condition than in the 2T condition, but bone nonunion remained in the 1T condition at the last follow-up. These results validate our model. Both quantitative and qualitative evaluations of bone regeneration are needed. Despite the assessment of bone consolidation previously described between 8 and 12 weeks [22, 25, 26], we believe that it is better to wait beyond 24 weeks to look for complete cortical consolidation, as we did. This was not evidenced in previous models [22, 25, 26].

This model is interesting for the evaluation of bone substitutes. The osteosynthesis plate is not deleterious for the evaluation of biomaterials [29], and a 4-mm defect allows the use of a limited amount of material. Furthermore, in the two-stage conditions compared to the one-stage conditions, the differences in bone regeneration were greater between the empty condition and the control conditions or with a syngeneic graft. This can be explained by the presence of the spacer during the first stage, which prevents spontaneous bone healing and osteoconduction along the plate.

Despite its dual osteoconductive and osteoinductive capacity [40], GlassBone alone was not sufficient to consolidate the bone defect. Bone volume was greater in the bone defects of the rats in the 2T-BV group than in those in the 1T-BV group. The IM alone supports bone healing by helping to revascularize the bone graft and prevent its resorption, as suggested by Pelissier et al. [14]. This is probably also the case for a biomaterial with an IM that contains the material and prevents leakage into the surrounding soft tissue.

On histomorphological analysis, areas of ossification contacted the bioactive glass. These results are encouraging and require further investigation with the concomitant use of syngeneic grafts and bioactive glass. Two clinical studies [41, 42] have evaluated this biomaterial in spinal surgery. When GlassBone was combined with a bone autograft, the results were comparable to those obtained with an autograft alone. This could improve the rate and duration of consolidation or decrease the amount of bone grafting needed, which is often limited in clinical practice [11].

## 4. Conclusions

Under the limitations of animal welfare and the practicality of a cost-effective model, our results demonstrate the versatility, reproducibility and functionality of this model to evaluate bone regeneration with the use of a biomaterial in the IM technique.

## Data Availability

The data that support the findings of this study are available in the Supporting Information of this article.

## Supporting Information

Figure S1: surgical procedure; Figure S2: Morton and Griffith score [32]; Figure S3: weight of the rats over time after the first surgical; Figure S4: weight of the rats over time after the second surgical procedure; Figure S5: Morton and Griffith score [32] after the first surgical procedure; Figure S6: Morton and Griffith score [32] after the second surgical procedure; Figure S7: CT radiological control after cutting procedures at 6 weeks; Figure S8: CT radiological control after piezotome osteotomies with the two osteosynthesis devices.

## References

[1] Gugala Z., Gogolewski S. (1999). Regeneration of Segmental Diaphyseal Defects in Sheep Tibiae Using Resorbable Polymeric Membranes: A Preliminary Study. *Journal of Orthopaedic Trauma*.

[2] Schmitz J. P., Hollinger J. O. (1986). The Critical Size Defect as an Experimental Model for Craniomandibulofacial Nonunions. *Clinical Orthopaedics and Related Research*.

[3] Wiese A., Pape H. C. (2010). Bone Defects Caused by High-Energy Injuries, Bone Loss, Infected Nonunions, and Nonunions. *Orthopedic Clinics of North America*.

[4] Rustom L. E., Poellmann M. J., Wagoner Johnson A. J. (2019). Mineralization in Micropores of Calcium Phosphate Scaffolds. *Acta Biomaterialia*.

[5] Han C. S., Wood M. B., Bishop A. T., Cooney W. P. (1992). Vascularized Bone Transfer. *Journal of Bone and Joint Surgery*.

[6] May J. W., Jupiter J. B., Weiland A. J., Byrd H. S. (1989). Clinical Classification of Post-traumatic Tibial Osteomyelitis. *Journal of Bone and Joint Surgery*.

[7] Masquelet A. C. (2003). Muscle Reconstruction in Reconstructive Surgery: Soft Tissue Repair and Long Bone Reconstruction. *Langenbeck’s Archives of Surgery*.

[8] Goldstein R. Y., Jordan C. J., McLaurin T. M., Grant A. (2013). The Evolution of the Ilizarov Technique: Part 2: The Principles of Distraction Osteosynthesis. *Bulletin of the Hospital for Joint Diseases*.

[9] Allsopp B. J., Hunter-Smith D. J., Rozen W. M. (2016). Vascularized versus Nonvascularized Bone Grafts: What Is the Evidence?. *Clinical Orthopaedics and Related Research*.

[10] Masquelet A. C., Fitoussi F., Begue T., Muller G. P. (2000). Reconstruction of the Long Bones by the Induced Membrane and Spongy Autograft. *Annales de Chirurgie Plastique et Esthetique*.

[11] Lasanianos N. G., Kanakaris N. K., Giannoudis P. V. (2010). Current Management of Long Bone Large Segmental Defects. *Orthopedie Traumatologie*.

[12] Masquelet A., Kanakaris N. K., Obert L., Stafford P., Giannoudis P. V. (2019). Bone Repair Using the Masquelet Technique. *Journal of Bone and Joint Surgery*.

[13] Pelissier P., Bollecker V., Martin D., Baudet J. (2002). Reconstruction du pied par la technique « Bi-Masquelet. *Annales de Chirurgie Plastique et Esthetique*.

[14] Pelissier P., Masquelet A., Bareille R., Pelissier S. M., Amedee J. (2004). Induced Membranes Secrete Growth Factors Including Vascular and Osteoinductive Factors and Could Stimulate Bone Regeneration. *Journal of Orthopaedic Research*.

[15] Viateau V., Guillemin G., Calando Y. (2006). Induction of a Barrier Membrane to Facilitate Reconstruction of Massive Segmental Diaphyseal Bone Defects: An Ovine Model. *Veterinary Surgery*.

[16] Mathieu L., Murison J. C., de Rousiers A. (2021). The Masquelet Technique: Can Disposable Polypropylene Syringes Be an Alternative to Standard PMMA Spacers? A Rat Bone Defect Model. *Clinical Orthopaedics and Related Research*.

[17] Gouron R., Petit L., Boudot C. (2017). Osteoclasts and Their Precursors Are Present in the Induced‐membrane during Bone Reconstruction Using the Masquelet Technique. *Journal of tissue engineering and regenerative medicine*.

[18] Gruber H. E., Riley F. E., Hoelscher G. L. (2012). Osteogenic and Chondrogenic Potential of Biomembrane Cells from the PMMA-Segmental Defect Rat Model. *Journal of Orthopaedic Research*.

[19] Gruber H. E., Gettys F. K., Montijo H. E. (2013). Genomewide Molecular and Biologic Characterization of Biomembrane Formation Adjacent to a Methacrylate Spacer in the Rat Femoral Segmental Defect Model. *Journal of Orthopaedic Trauma*.

[20] Henrich D., Seebach C., Nau C. (2016). Establishment and Characterization of the Masquelet Induced Membrane Technique in a Rat Femur Critical-Sized Defect Model. *J Tissue Eng Regen Med*.

[21] Nau C., Seebach C., Trumm A. (2016). Alteration of Masquelet’s Induced Membrane Characteristics by Different Kinds of Antibiotic Enriched Bone Cement in a Critical Size Defect Model in the Rat’s Femur. *Injury*.

[22] Tang Q., Jin H., Tong M. (2018). Inhibition of Dll4/Notch1 Pathway Promotes Angiogenesis of Masquelet’s Induced Membrane in Rats. *Experimental and Molecular Medicine*.

[23] Tang Q., Tong M., Zheng G., Shen L., Shang P., Liu H. (2018). Masquelet’s Induced Membrane Promotes the Osteogenic Differentiation of Bone Marrow Mesenchymal Stem Cells by Activating the Smad and MAPK Pathways. *Am J Transl Res*.

[24] Bosemark P., Perdikouri C., Pelkonen M., Isaksson H., Tägil M. (2015). The Masquelet Induced Membrane Technique with BMP and a Synthetic Scaffold Can Heal a Rat Femoral Critical Size Defect. *Journal of Orthopaedic Research*.

[25] Li W., Zara J. N., Siu R. K. (2011). Nell-1 Enhances Bone Regeneration in a Rat Critical-Sized Femoral Segmental Defect Model. *Plastic and Reconstructive Surgery*.

[26] Nau C., Simon S., Schaible A. (2018). Influence of the Induced Membrane Filled with Syngeneic Bone and Regenerative Cells on Bone Healing in a Critical Size Defect Model of the Rat’s Femur. *Injury*.

[27] Ma Y., Jiang N., Zhang X. (2018). Calcium Sulfate Induced versus PMMA-Induced Membrane in a Critical-Sized Femoral Defect in a Rat Model. *Scientific Reports*.

[28] Schindeler A., Mills R. J., Bobyn J. D., Little D. G. (2018). Preclinical Models for Orthopedic Research and Bone Tissue Engineering. *Journal of Orthopaedic Research*.

[29] Klein C., Monet M., Barbier V. (2020). The Masquelet Technique: Current Concepts, Animal Models, and Perspectives. *J Tissue Eng Regen Med*.

[30] Jerome C., Hoch B., Carlson C. S., Treuting P. M., Dintzis S. M., Montine K. S. (2018). 5: Skeletal System. *Comparative Anatomy and Histology*.

[31] Charbonnier B., Manassero M., Bourguignon M. (2020). Custom-made Macroporous Bioceramic Implants Based on Triply-Periodic Minimal Surfaces for Bone Defects in Load-Bearing Sites. *Acta Biomaterialia*.

[32] Morton D. B., Griffiths P. H. (1985). Guidelines on the Recognition of Pain, Distress and Discomfort in Experimental Animals and an Hypothesis for Assessment. *The Veterinary Record*.

[33] Schindelin J., Arganda-Carreras I., Frise E. (2012). Fiji: An Open-Source Platform for Biological-Image Analysis. *Nature Methods*.

[34] Pearce A. I., Richards R. G., Milz S., Schneider E., Pearce S. G. (2007). Animal Models for Implant Biomaterial Research in Bone: A Review. *European Cells and Materials*.

[35] Leclercq P., Dohan D. (2004). De l’intérêt du bistouri ultrasonore en implantologie: Technologies, applications cliniques: 1re partie: Technologies. *Implantodontie*.

[36] Vercellotti T. (2004). Technological Characteristics and Clinical Indications of Piezoelectric Bone Surgery. *Minerva Stomatologica*.

[37] Horton J. E., Tarpley T. M., Wood L. D. (1975). The Healing of Surgical Defects in Alveolar Bone Produced with Ultrasonic Instrumentation, Chisel, and Rotary Bur. *Oral Surgery, Oral Medicine, Oral Pathology*.

[38] Aurégan J. C., Bégué T. (2014). Induced Membrane for Treatment of Critical Sized Bone Defect: A Review of Experimental and Clinical Experiences. *International Orthopaedics*.

[39] Aurégan J. C., Bégué T., Rigoulot G., Glorion C., Pannier S. (2016). Success Rate and Risk Factors of Failure of the Induced Membrane Technique in Children: A Systematic Review. *Injury*.

[40] Rizwan M., Hamdi M., Basirun W. J. (2017). Bioglass® 45S5-Based Composites for Bone Tissue Engineering and Functional Applications. *Journal of Biomedical Materials Research Part A*.

[41] Barrey C., Broussolle T. (2019). Clinical and Radiographic Evaluation of Bioactive Glass in Posterior Cervical and Lumbar Spinal Fusion. *European Journal of Orthopaedic Surgery and Traumatology*.

[42] Ilharreborde B., Morel E., Fitoussi F. (2008). Bioactive Ggass as a Bone Substitute for Spinal Fusion in Adolescent Idiopathic Scoliosis: A Comparative Study with Iliac Crest Autograft. *Journal of Pediatric Orthopaedics*.

