# Supplementary material for: Optimisation and Validation of an Induced Membrane Technique Model to Assess Bone Regeneration in Rats
Source: J Tissue Eng Regen Med. 2025 Apr 21;2025:7357277. doi: 10.1155/term/7357277 (PMC12037239; doi:10.1155/term/7357277)
Supplement: Supporting Information — Additional supporting information can be found online in the Supporting Information section. [file 7357277.f1.zip › Supplementary data Editable files.pptx]

## Slide 1
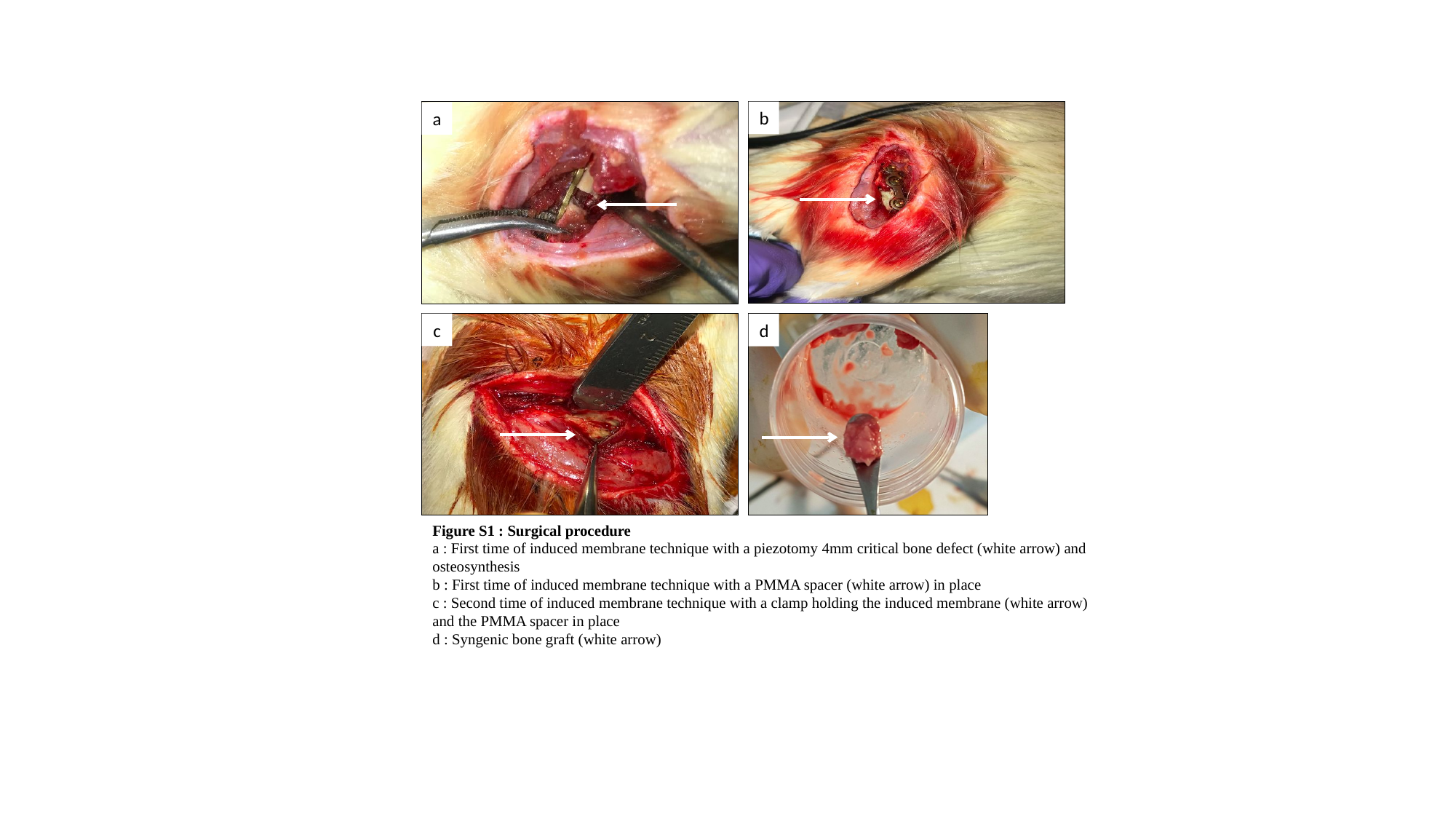

b
a
c
d
Figure S1 : Surgical procedure
a : First time of induced membrane technique with a piezotomy 4mm critical bone defect (white arrow) and osteosynthesis
b : First time of induced membrane technique with a PMMA spacer (white arrow) in place
c : Second time of induced membrane technique with a clamp holding the induced membrane (white arrow) and the PMMA spacer in place
d : Syngenic bone graft (white arrow)

## Slide 2
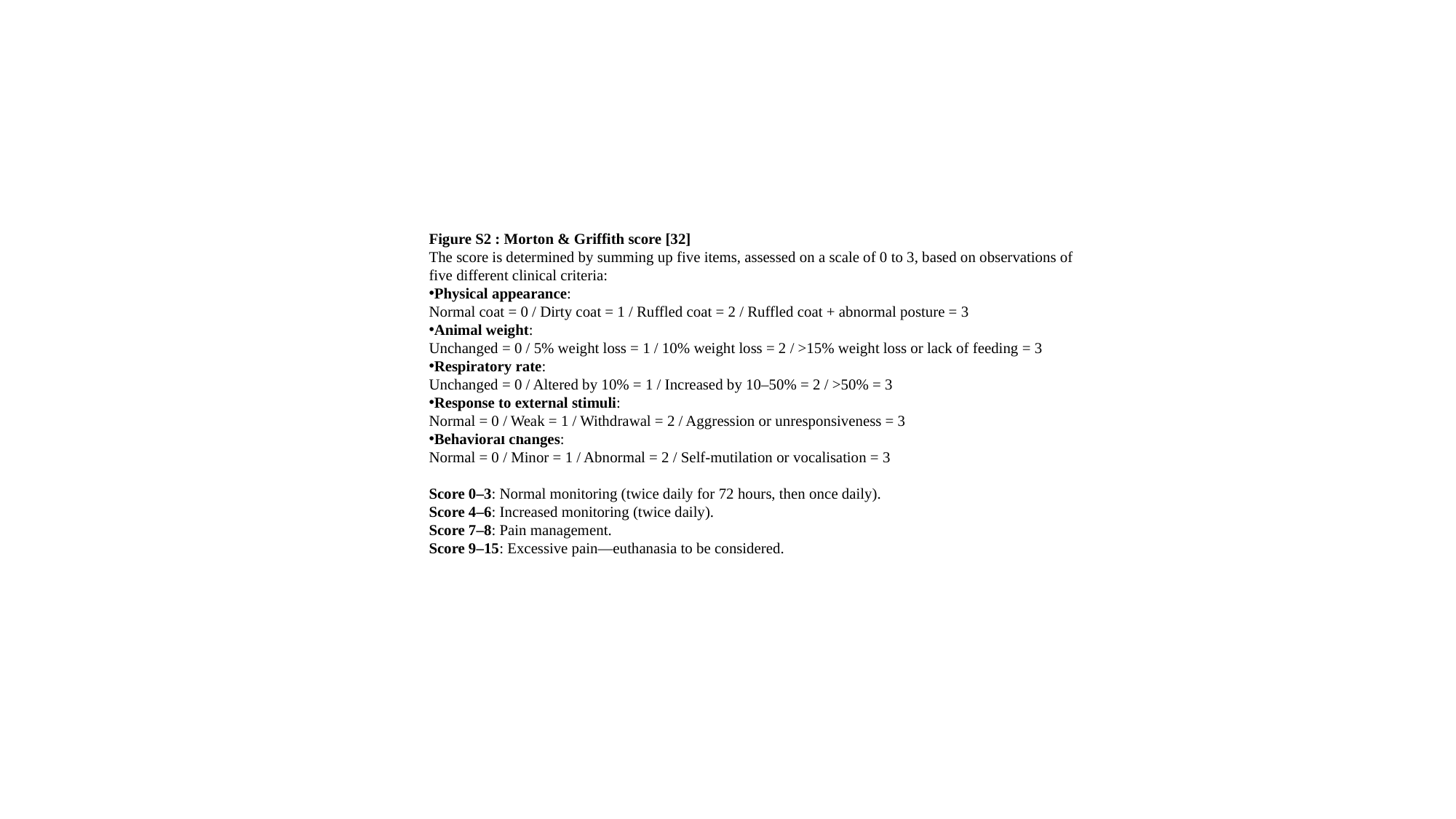

Figure S2 : Morton & Griffith score [32]
The score is determined by summing up five items, assessed on a scale of 0 to 3, based on observations of five different clinical criteria:
Physical appearance:
Normal coat = 0 / Dirty coat = 1 / Ruffled coat = 2 / Ruffled coat + abnormal posture = 3
Animal weight:
Unchanged = 0 / 5% weight loss = 1 / 10% weight loss = 2 / >15% weight loss or lack of feeding = 3
Respiratory rate:
Unchanged = 0 / Altered by 10% = 1 / Increased by 10–50% = 2 / >50% = 3
Response to external stimuli:
Normal = 0 / Weak = 1 / Withdrawal = 2 / Aggression or unresponsiveness = 3
Behavioral changes:
Normal = 0 / Minor = 1 / Abnormal = 2 / Self-mutilation or vocalisation = 3
Score 0–3: Normal monitoring (twice daily for 72 hours, then once daily).
Score 4–6: Increased monitoring (twice daily).
Score 7–8: Pain management.
Score 9–15: Excessive pain—euthanasia to be considered.

## Slide 3
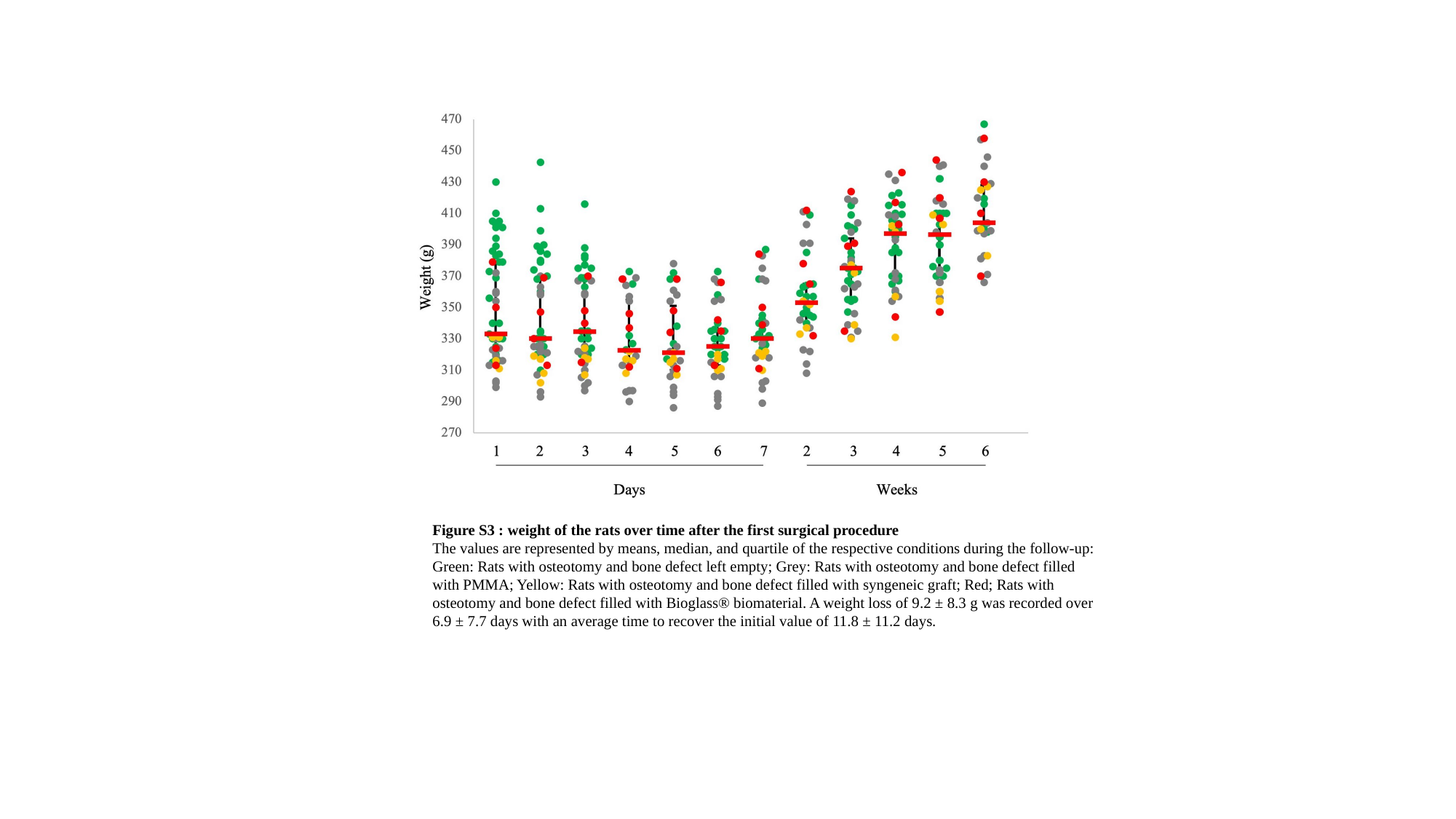

Figure S3 : weight of the rats over time after the first surgical procedure
The values are represented by means, median, and quartile of the respective conditions during the follow-up:
Green: Rats with osteotomy and bone defect left empty; Grey: Rats with osteotomy and bone defect filled with PMMA; Yellow: Rats with osteotomy and bone defect filled with syngeneic graft; Red; Rats with osteotomy and bone defect filled with Bioglass® biomaterial. A weight loss of 9.2 ± 8.3 g was recorded over 6.9 ± 7.7 days with an average time to recover the initial value of 11.8 ± 11.2 days.

## Slide 4
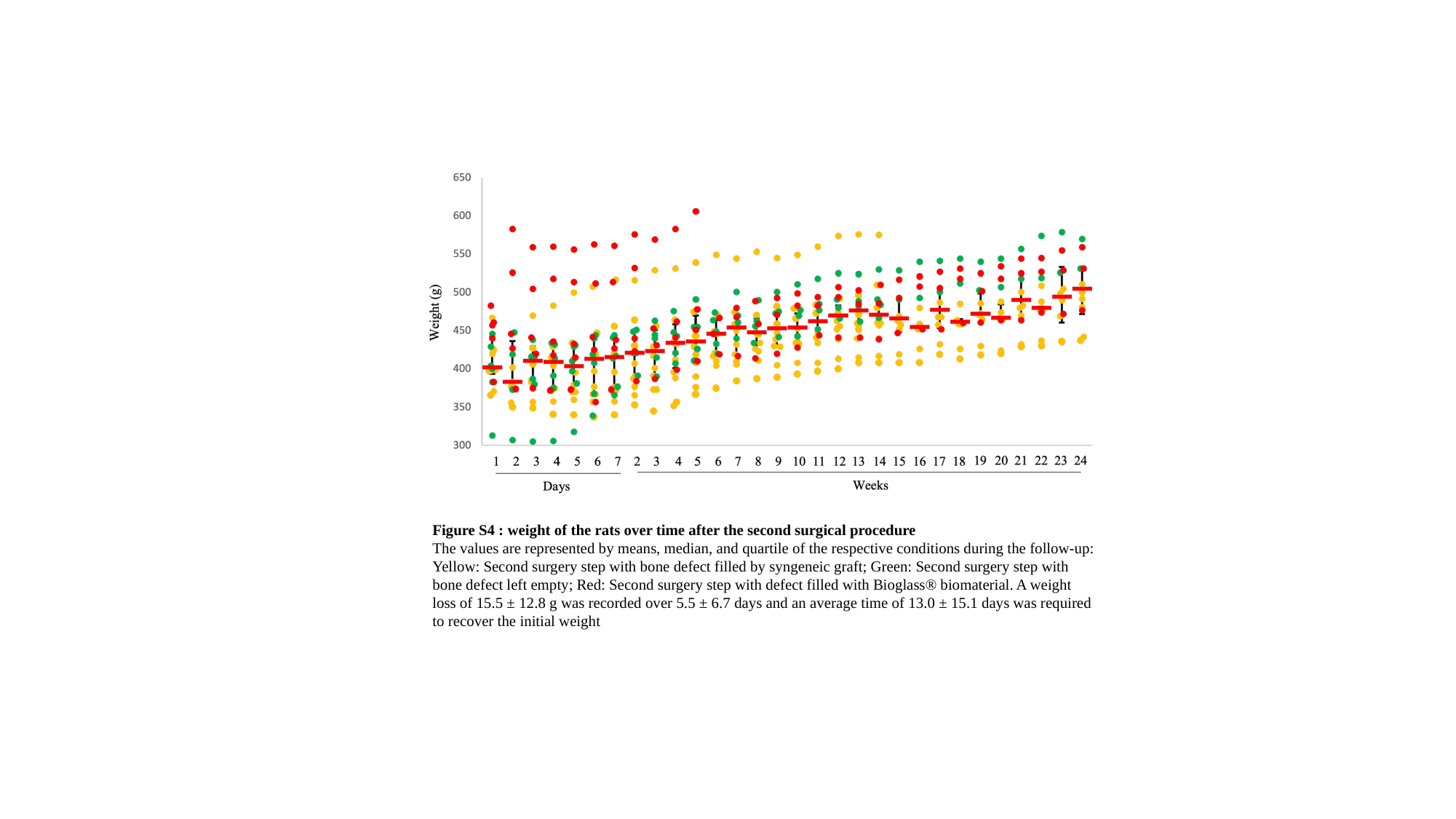

Figure S4 : weight of the rats over time after the second surgical procedure
The values are represented by means, median, and quartile of the respective conditions during the follow-up:
Yellow: Second surgery step with bone defect filled by syngeneic graft; Green: Second surgery step with bone defect left empty; Red: Second surgery step with defect filled with Bioglass® biomaterial. A weight loss of 15.5 ± 12.8 g was recorded over 5.5 ± 6.7 days and an average time of 13.0 ± 15.1 days was required to recover the initial weight

## Slide 5
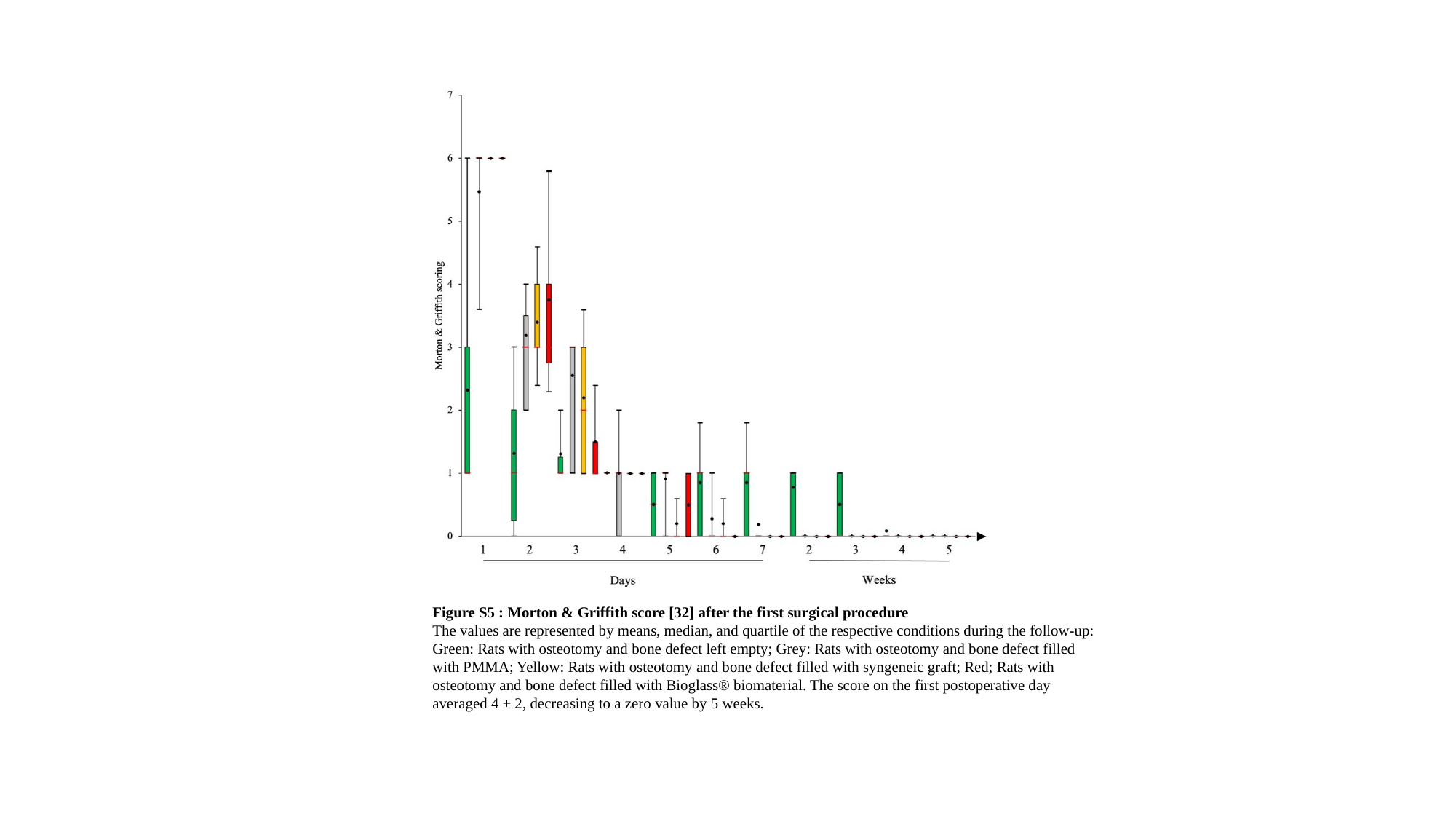

Figure S5 : Morton & Griffith score [32] after the first surgical procedure
The values are represented by means, median, and quartile of the respective conditions during the follow-up:
Green: Rats with osteotomy and bone defect left empty; Grey: Rats with osteotomy and bone defect filled with PMMA; Yellow: Rats with osteotomy and bone defect filled with syngeneic graft; Red; Rats with osteotomy and bone defect filled with Bioglass® biomaterial. The score on the first postoperative day averaged 4 ± 2, decreasing to a zero value by 5 weeks.

## Slide 6
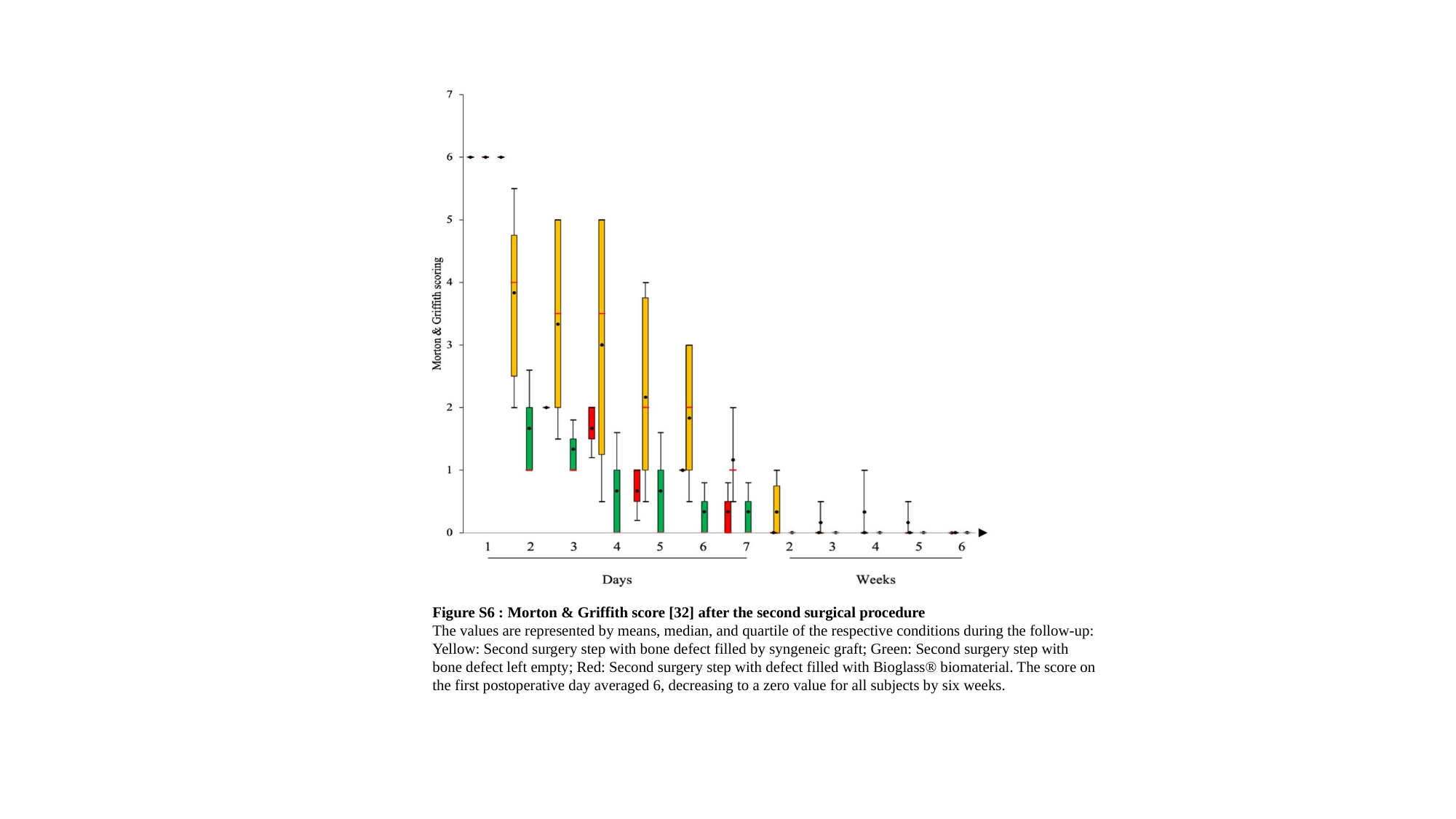

Figure S6 : Morton & Griffith score [32] after the second surgical procedure
The values are represented by means, median, and quartile of the respective conditions during the follow-up:
Yellow: Second surgery step with bone defect filled by syngeneic graft; Green: Second surgery step with bone defect left empty; Red: Second surgery step with defect filled with Bioglass® biomaterial. The score on the first postoperative day averaged 6, decreasing to a zero value for all subjects by six weeks.

## Slide 7
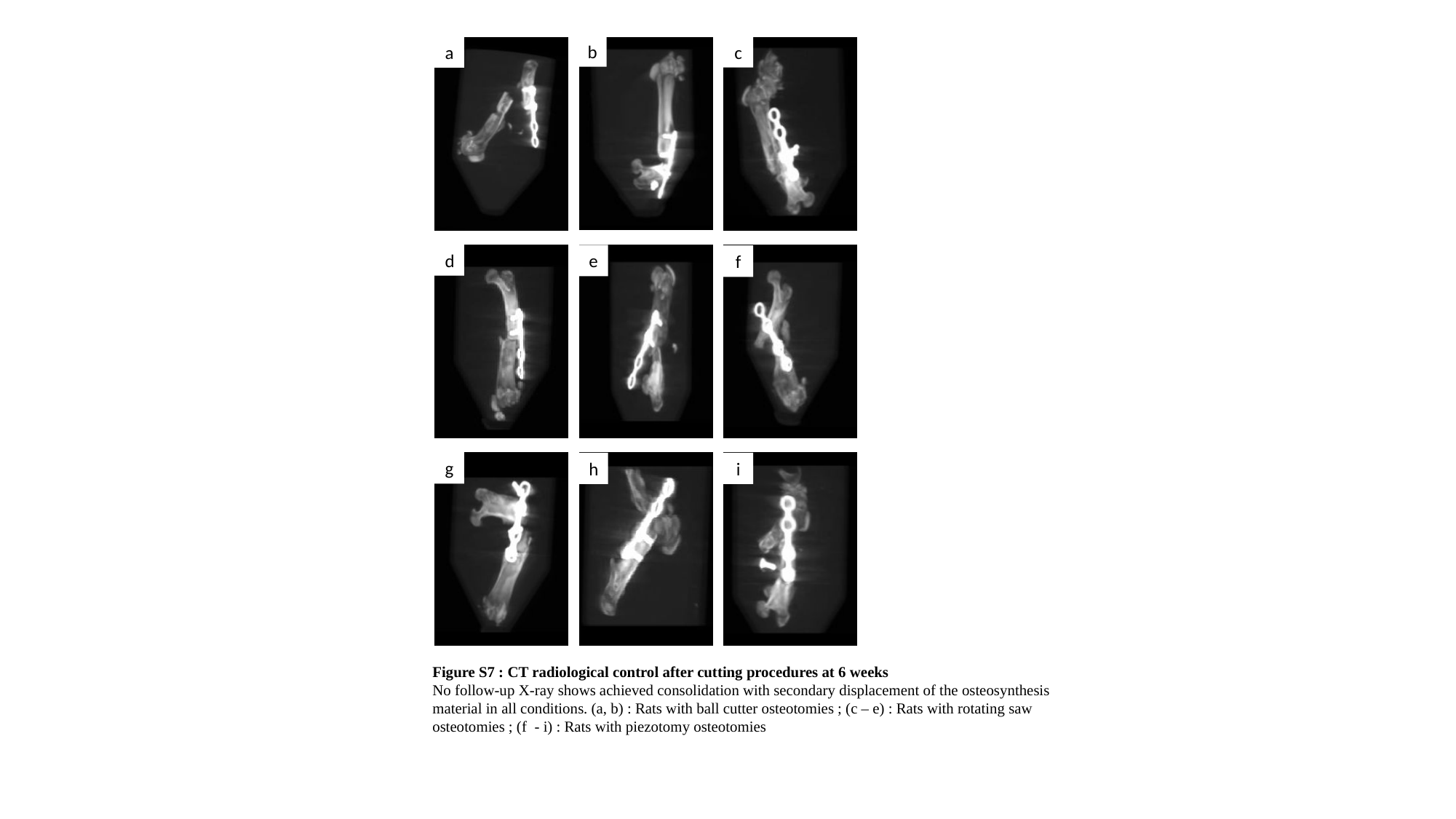

b
c
a
d
e
f
g
h
i
Figure S7 : CT radiological control after cutting procedures at 6 weeks
No follow-up X-ray shows achieved consolidation with secondary displacement of the osteosynthesis material in all conditions. (a, b) : Rats with ball cutter osteotomies ; (c – e) : Rats with rotating saw osteotomies ; (f - i) : Rats with piezotomy osteotomies

## Slide 8
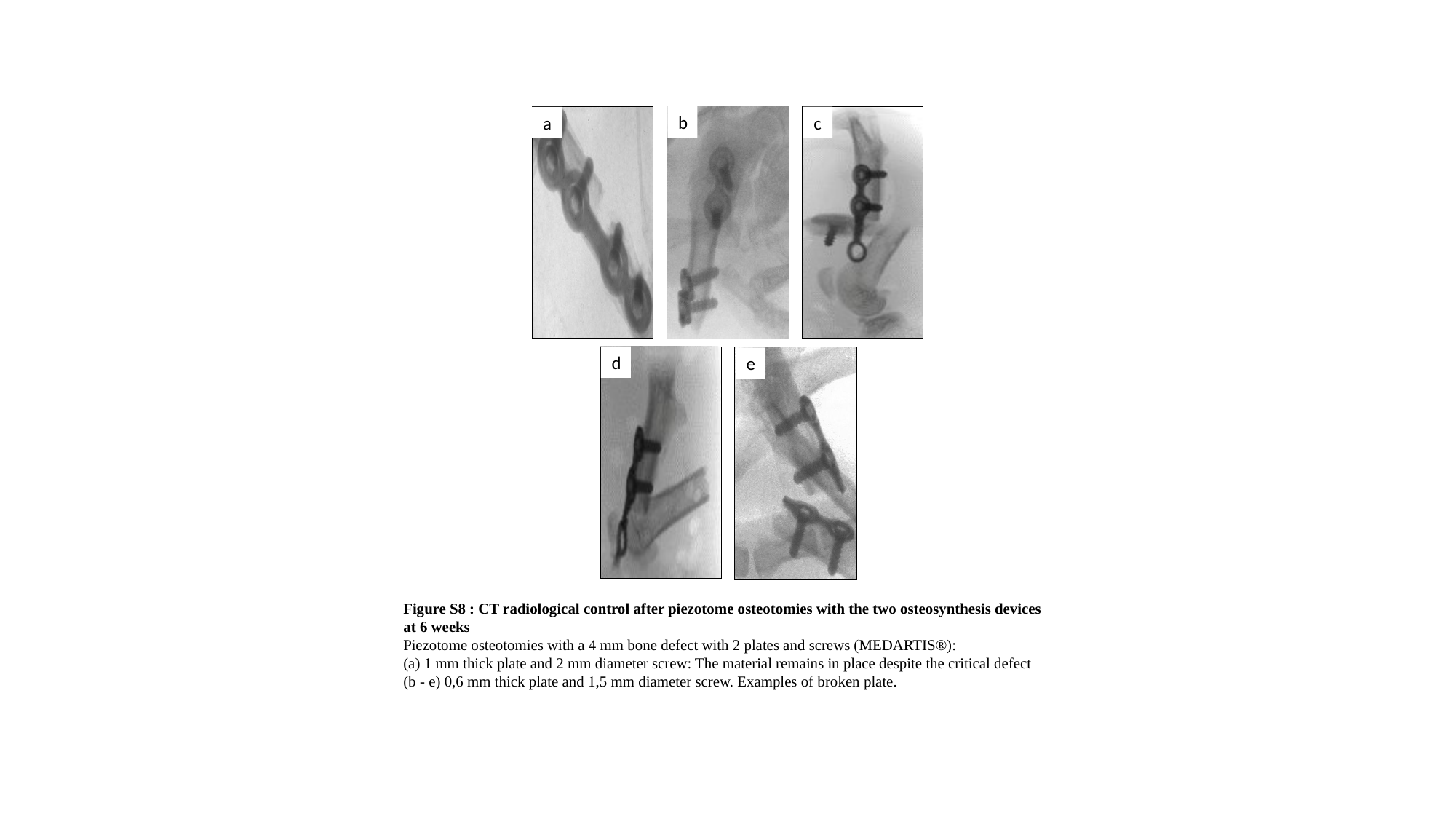

b
c
a
d
e
Figure S8 : CT radiological control after piezotome osteotomies with the two osteosynthesis devices at 6 weeks
Piezotome osteotomies with a 4 mm bone defect with 2 plates and screws (MEDARTIS®):
(a) 1 mm thick plate and 2 mm diameter screw: The material remains in place despite the critical defect
(b - e) 0,6 mm thick plate and 1,5 mm diameter screw. Examples of broken plate.
