# Supplementary figures and images for: Optimisation and Validation of an Induced Membrane Technique Model to Assess Bone Regeneration in Rats
Source: J Tissue Eng Regen Med. 2025 Apr 21;2025:7357277. doi: 10.1155/term/7357277 (PMC12037239; doi:10.1155/term/7357277)

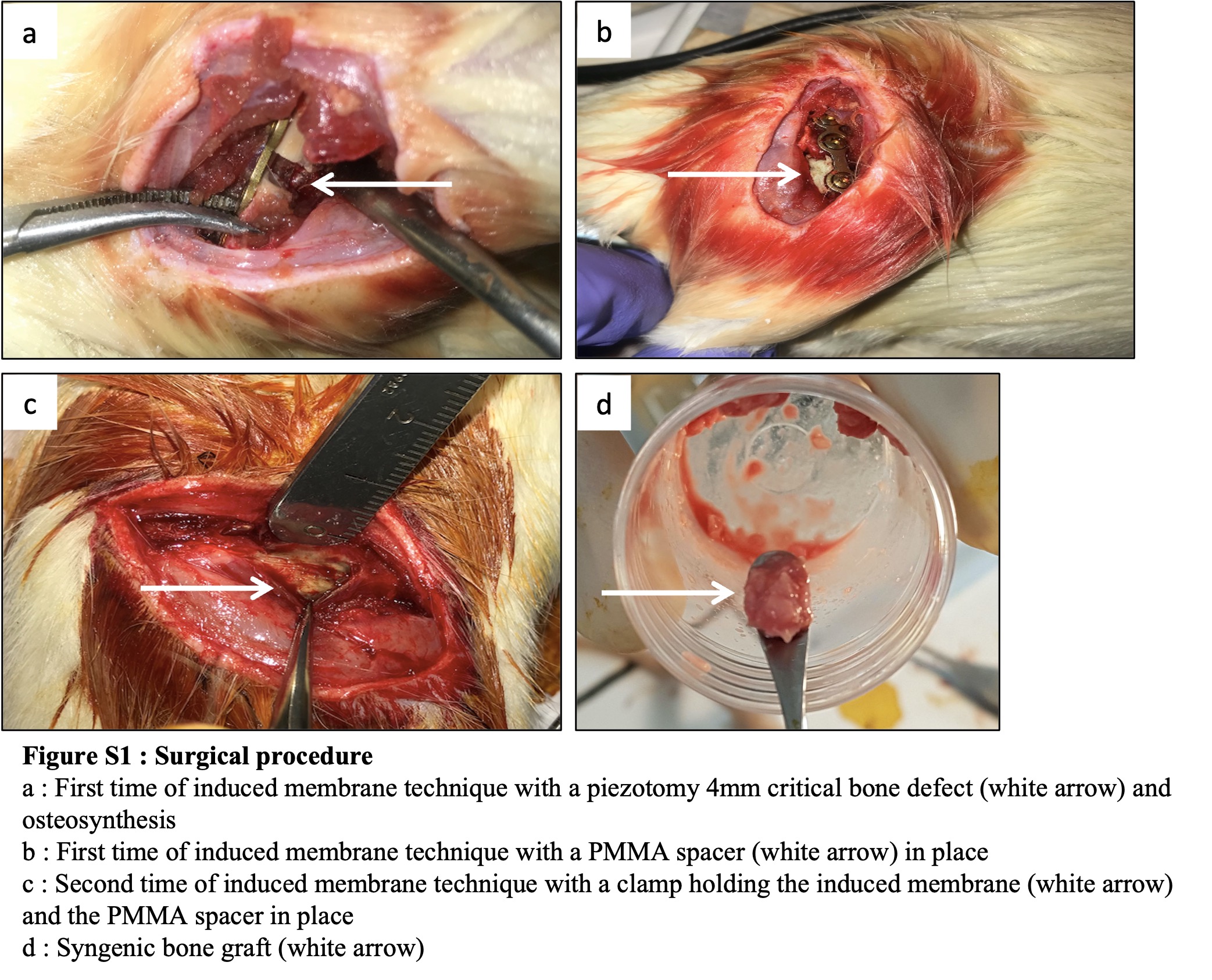

Supplement: Supporting Information — Additional supporting information can be found online in the Supporting Information section. [file 7357277.f1.zip › Supplementary data 1 R2.jpg]

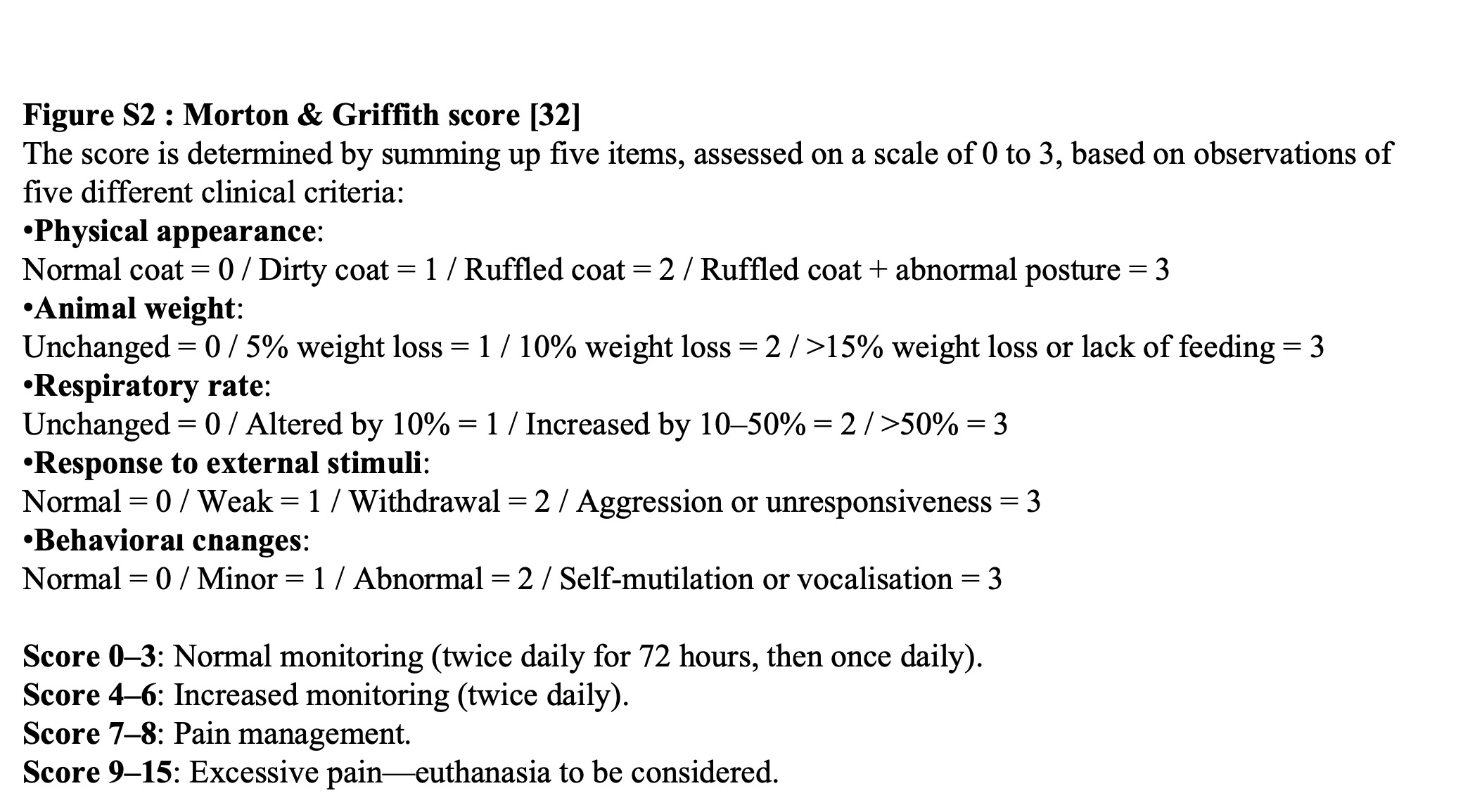

Supplement: Supporting Information — Additional supporting information can be found online in the Supporting Information section. [file 7357277.f1.zip › Supplementary data 2 R2.jpg]

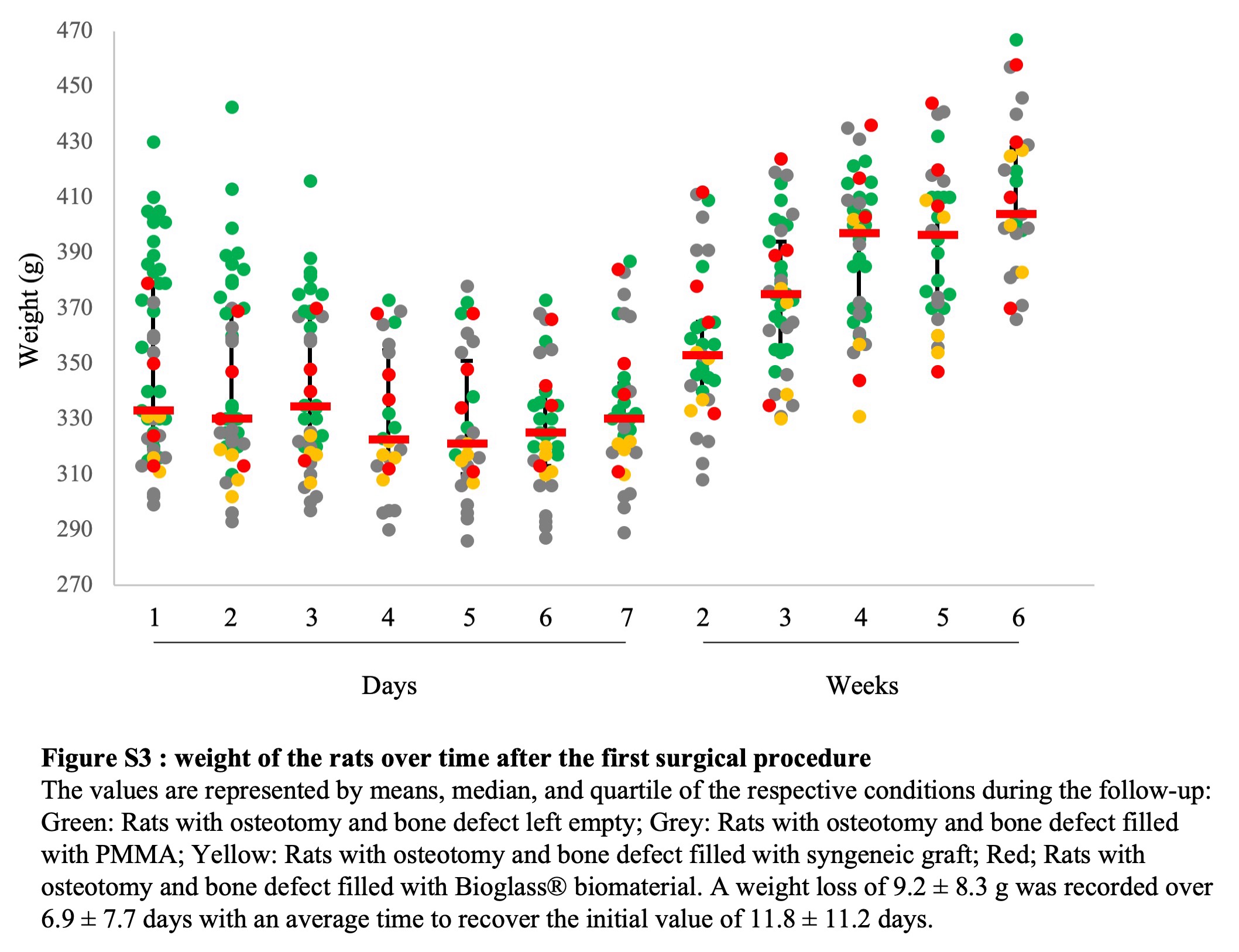

Supplement: Supporting Information — Additional supporting information can be found online in the Supporting Information section. [file 7357277.f1.zip › Supplementary data 3 R2.jpg]

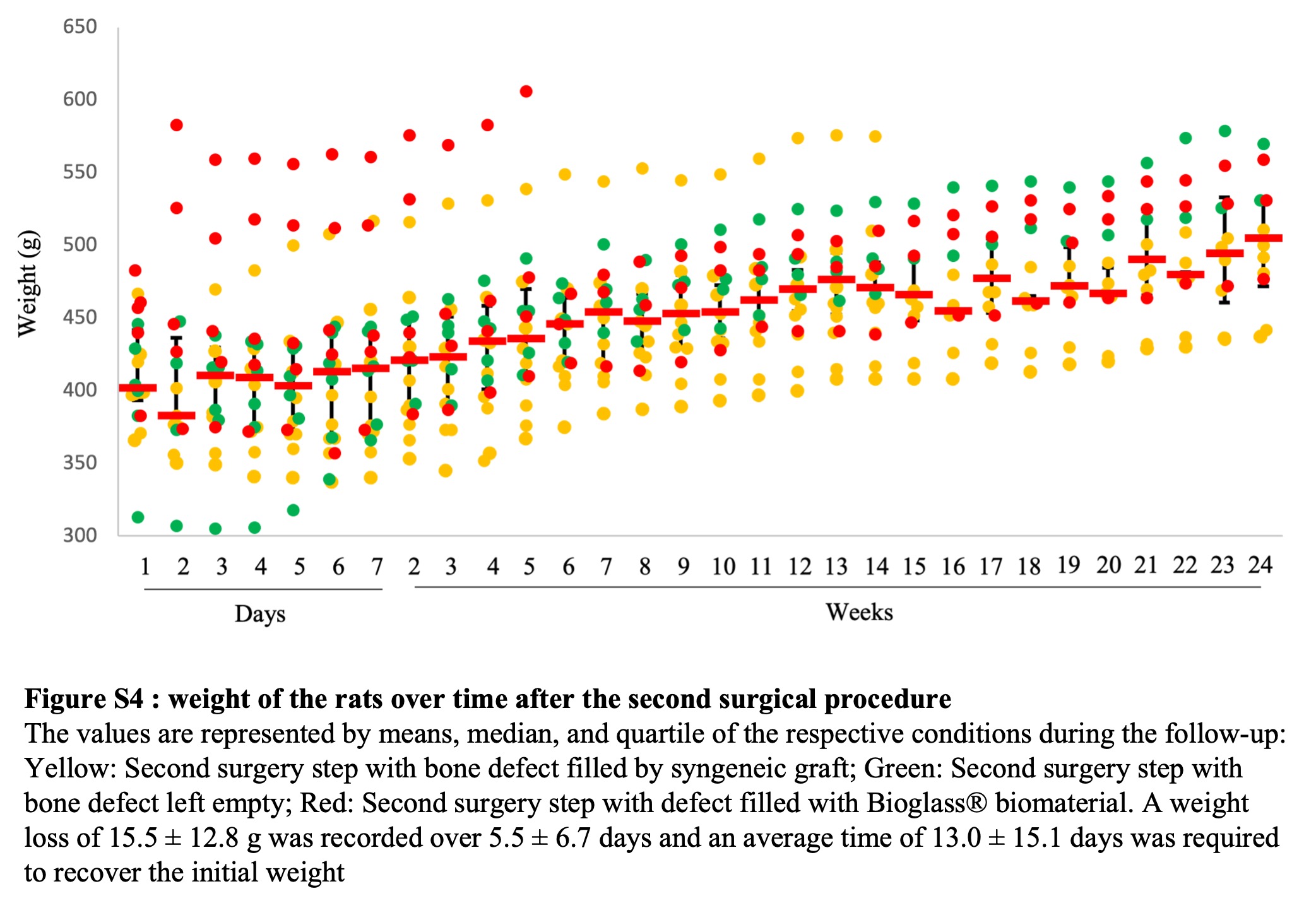

Supplement: Supporting Information — Additional supporting information can be found online in the Supporting Information section. [file 7357277.f1.zip › Supplementary data 4 R2.jpg]

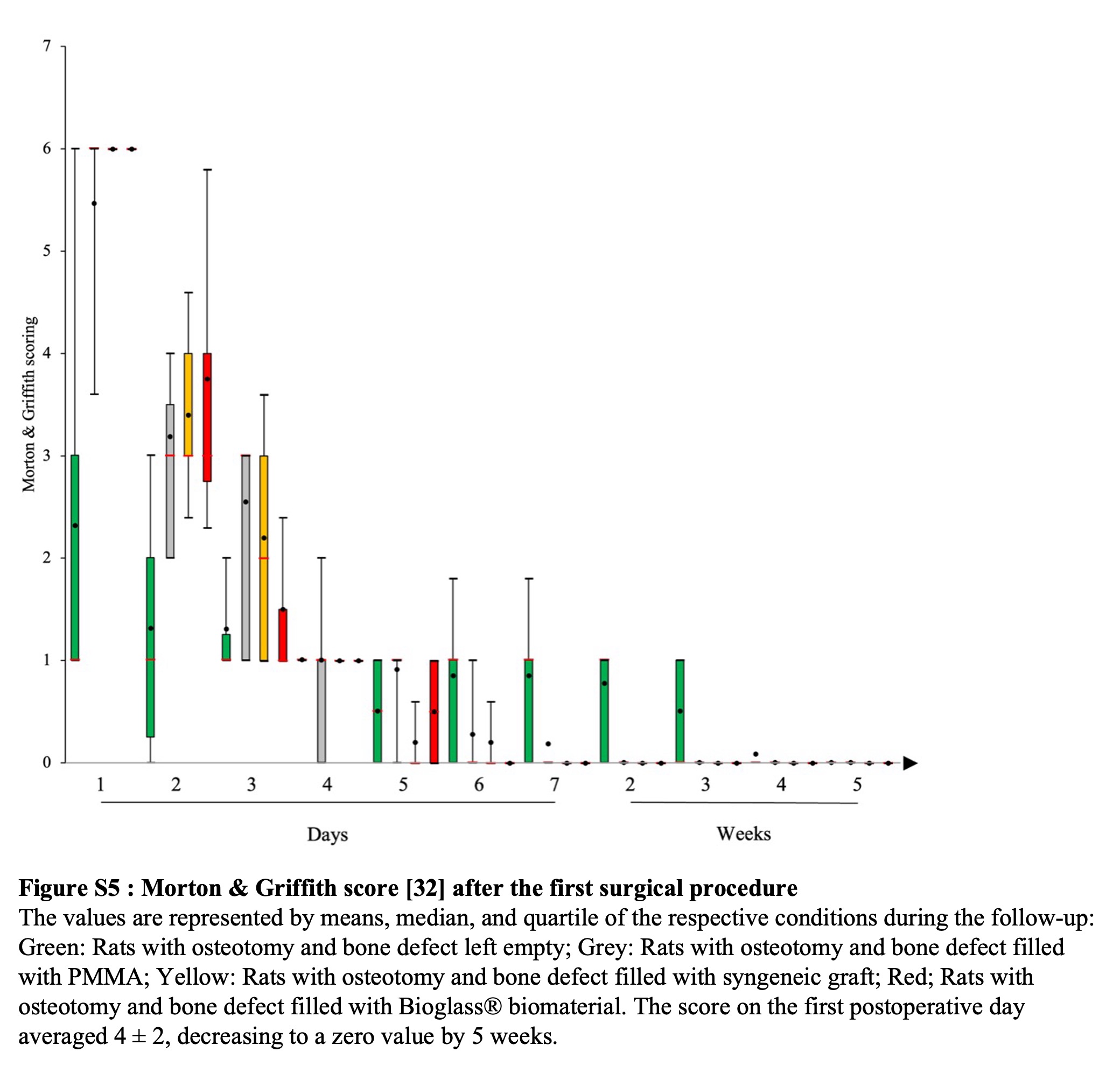

Supplement: Supporting Information — Additional supporting information can be found online in the Supporting Information section. [file 7357277.f1.zip › Supplementary data 5 R2.jpg]

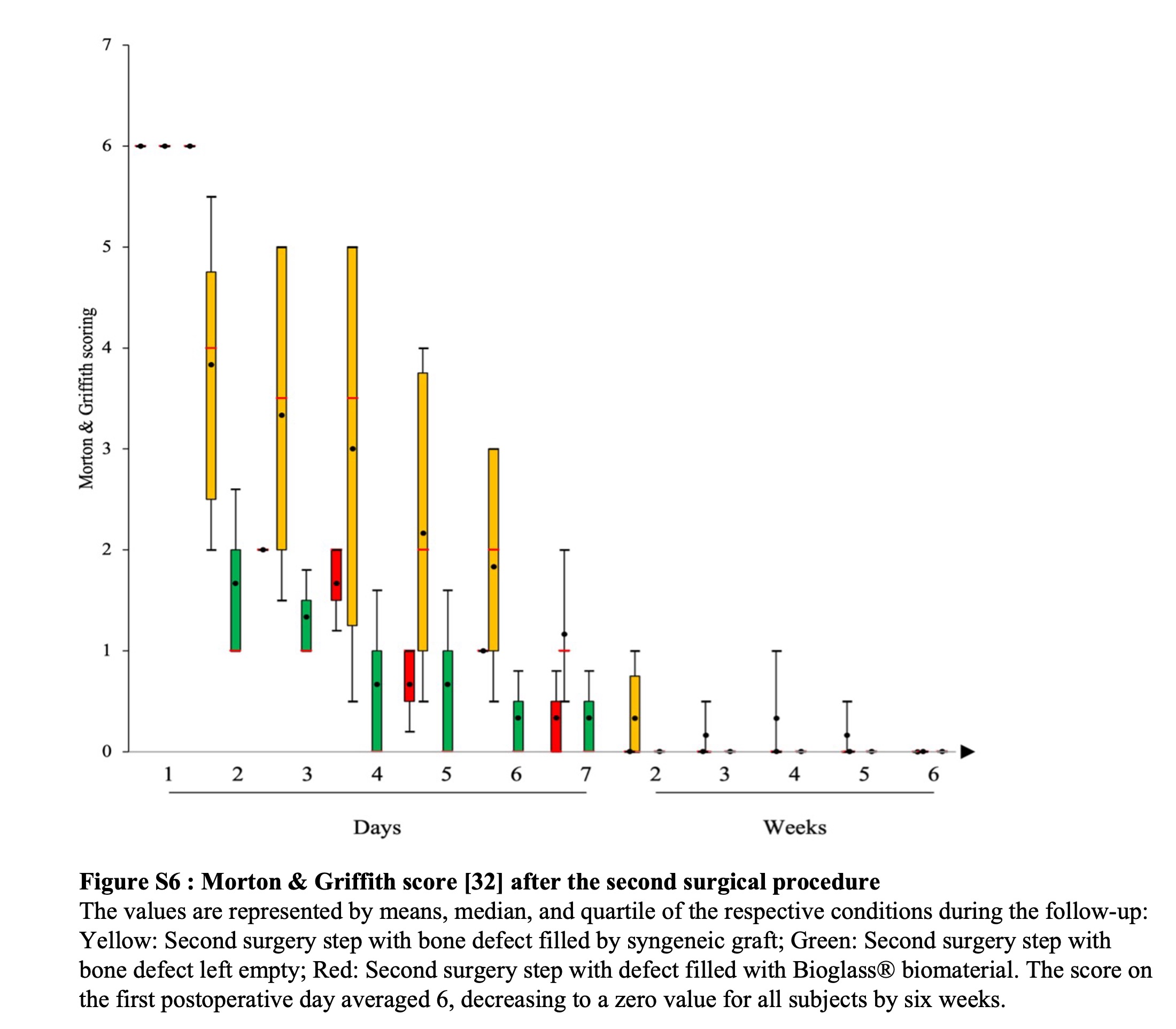

Supplement: Supporting Information — Additional supporting information can be found online in the Supporting Information section. [file 7357277.f1.zip › Supplementary data 6 R2.jpg]

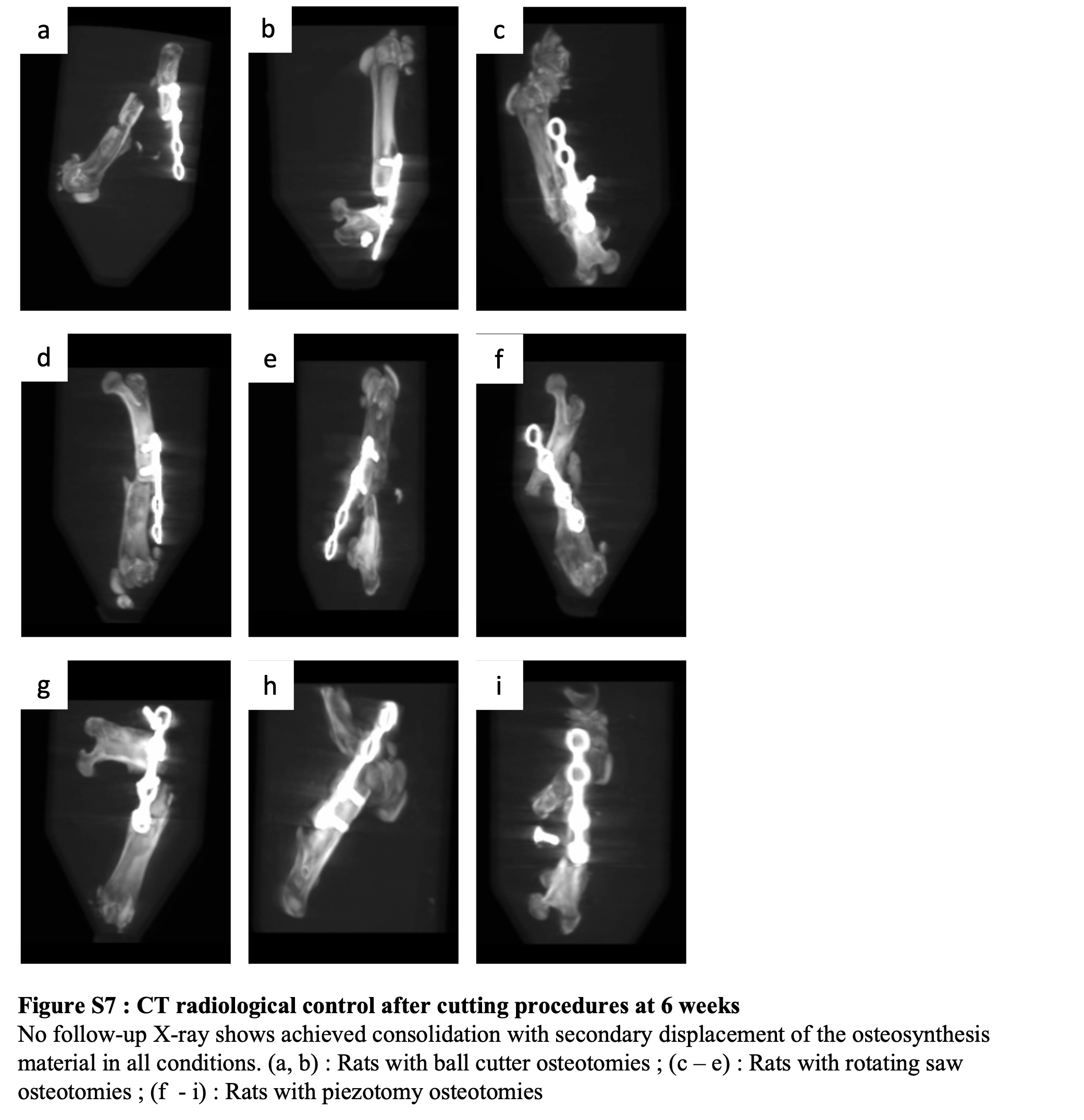

Supplement: Supporting Information — Additional supporting information can be found online in the Supporting Information section. [file 7357277.f1.zip › Supplementary data 7 R2.jpg]

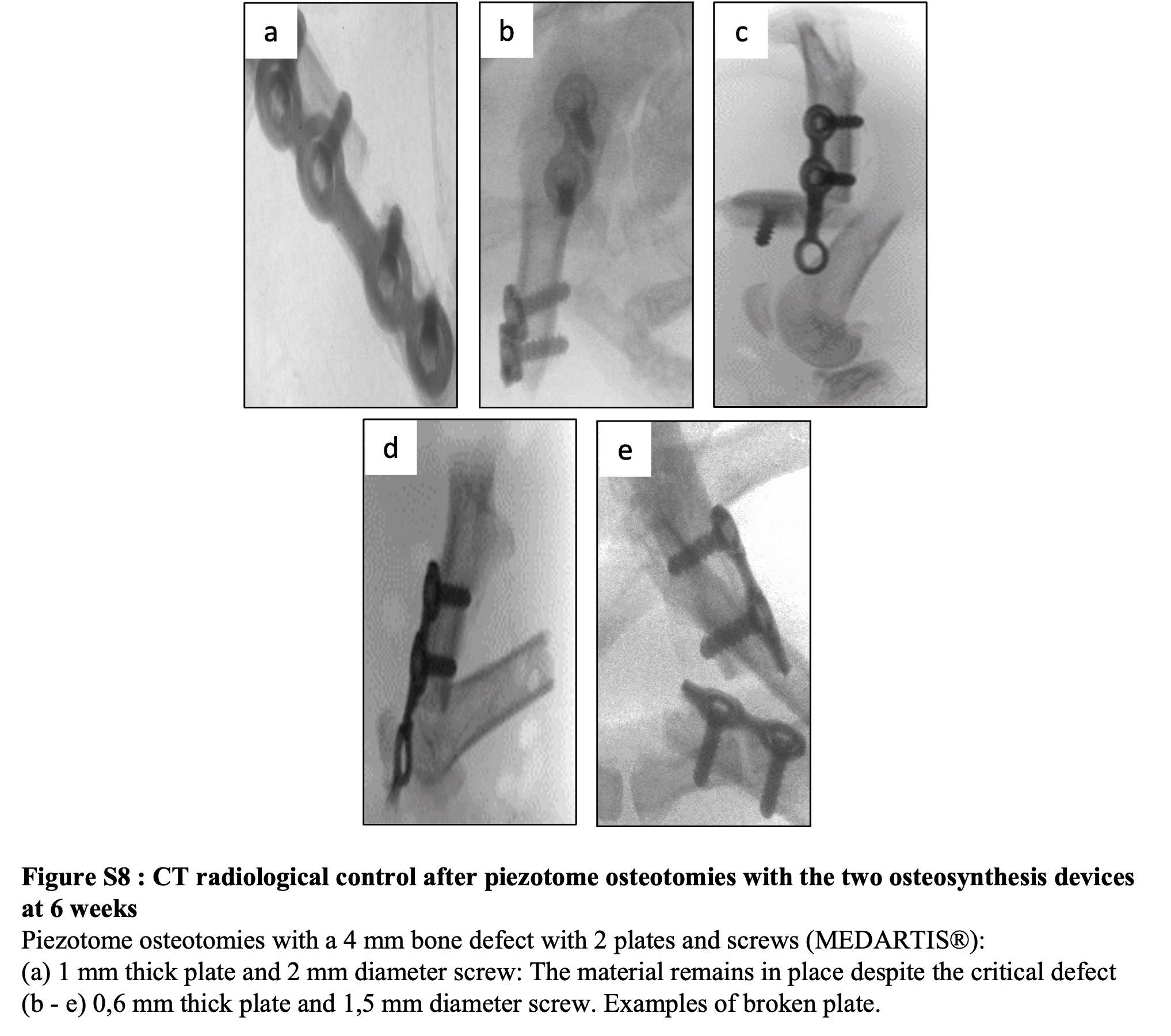

Supplement: Supporting Information — Additional supporting information can be found online in the Supporting Information section. [file 7357277.f1.zip › Supplementary data 8 R2.jpg]
